# One-pot Fluorination and Organocatalytic Robinson Annulation for Asymmetric Synthesis of Mono- and Difluorinated Cyclohexenones

**DOI:** 10.3390/molecules23092251

**Published:** 2018-09-04

**Authors:** Xin Huang, Weizhao Zhao, Xiaofeng Zhang, Miao Liu, Stanley N. S. Vasconcelos, Wei Zhang

**Affiliations:** 1 College of Chemistry and Life Sciences, Zhejiang Normal University, Jinhua 321004, China; zhao792129582@163.com; 2 Department of Chemistry, University of Massachusetts Boston, 100 Morrissey Boulevard, Boston, MA 02125, USA; Xiaofeng.Zhang002@umb.edu (X.Z.); liumiaomarcus@gmail.com (M.L.); 3 Departamento de Farmácia, Universidade de São Paulo, Av. Prof. Lineu Prestes, 580 São Paulo, SP 05508-000, Brazil; stanleynsv@gmail.com

**Keywords:** organocatalysis, fluorination, α-fluoro-β-keto ester, Michael addition, Robinson annulation, cyclohexenone

## Abstract

A one-pot fluorination and organocatalytic Robinson annulation sequence has been developed for asymmetric synthesis of 6-fluoroyclohex-2-en-1-ones and 4,6-difluorocyclohex-2-en-1-ones. The reactions promoted by cinchona alkaloid amine afforded products bearing two or three stereocenters in good to excellent yields with up to 99% ee and 20:1 dr.

## 1. Introduction

**Figure 1:**
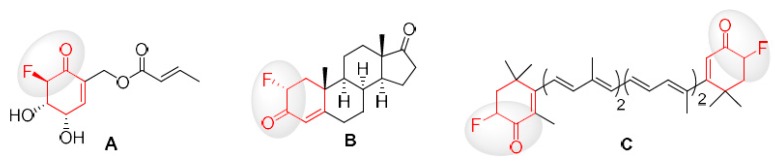
Bio-active fluorinated cyclohexenones.

The development of new synthetic methods for organofluorine compounds is an active topic in organic and medicinal chemistry. Introduction of fluorine atom(s) could have significant impact on molecules’ biological activity, bioavailability, and metabolic property [1,2,3,4]. Shown in Figure 1 are bioactive fluorinated cyclohexenones [5] including intermediate for antitumor agent COTC (Figure 1A) [6], aromatase inhibitor (Figure 1B) [7], and retinal protein bacteriorhodopsin (Figure 1C) [8].

The β-ketoester scaffold is a versatile synthon for being both electrophilic and nucleophilic sites [9]. The asymmetric Michael addition reactions of α-fluoro-β-keto esters [10,11,12,13,14,15] and other monofluorinated nucleophiles [16,17,18,19] is an attractive topic. These nucleophiles have been used to react with nitroolefins [10,11,13,14,16,17], *N*-alkyl maleimides [12,15], chalcones [18], and α,β-unsaturated aldehydes [19]. Beside the Michael addition reactions, some Michael addition-initiated reactions involving α-fluoro-β-keto esters have also been reported [20,21,22]. Those reactions include Michael/aldol (Robinson) [20], Michael/Michael/aldol [21], and Michael/aza-Henry/lactamization sequences [22] for the construction of cyclohexenones, cyclohexanones, and 2-piperidinones bearing multiple stereocenters.

**Scheme 1:**
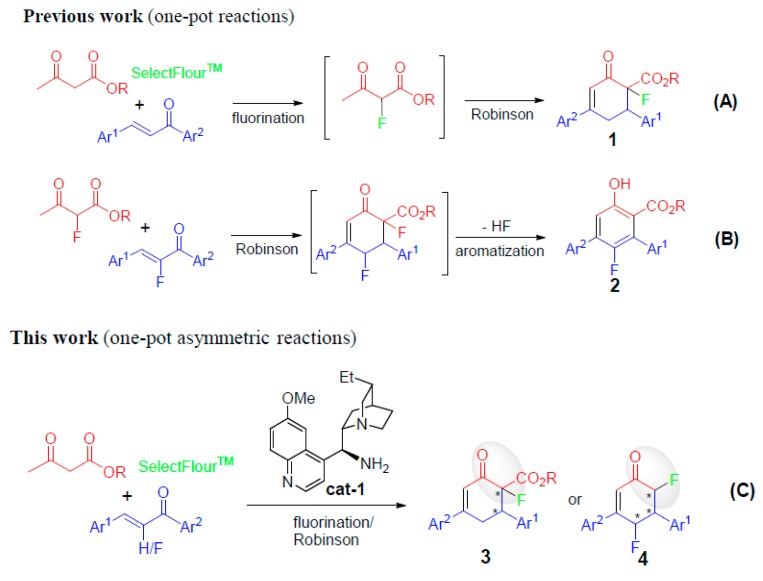
Robinson annulation-based one-pot synthesis.

As part of our recent effort on the synthesis of organofluorine compounds using α-fluoro-β-keto esters [23,24,25], we have reported a one-pot fluorination/Robinson annulation sequence for fluorinated cyclohexenones 1 (Scheme 1A) [23], Robinson annulation/dehydrofluorination/aromatization sequence for phenols 2 (Scheme 1B) [24], and pyridines [25]. We envisioned that in the presence of an organocatalyst, such as cinchona alkaloid primary amine **cat-1**, the one-pot asymmetric synthesis could be developed for the preparation of monofluorocyclohexenones 3 and difluorocyclohexenones 4 (Scheme 1C).

## 2. Results and Discussions

A number of six organocatalysts were screened for the asymmetric Robinson annulation reaction of chalcone **6a** and α-fluoro-β-ketoester **5a** (Table 1, entries 1–6). It was found that the reaction with 20 mol% of **cat-1** gave the desired product **3a** in 79% yield with 5:1 dr and 99% ee (entry 1), while other catalysts did not give good results. Acidity of the reaction system plays an important role in amine catalysis for lowering the LUMO energy of iminium ions [26]. Thus, an investigation of the reactions in the presence of different acids was carried out. The best result was achieved by adding CF_3_C_6_H_4_CO_2_H to afford **3a** in 89% yield with 9:1 dr and 99% ee (entry 10). We lowered the reaction temperature to −20 °C which slightly increased the dr to 12:1, but significantly decreased the yield to 51% (entry 13). Because we failed to get the crystal of **3** for X-ray structure analysis, the absolute configuration of **3a** was deduced based on the information reported in the literature [27]. The *S*-amine catalyst promoted a highly selective Re-face attack at the Michael acceptor. We believed that our reactions also catalyzed by the *S*-amine **cat-1** could have the same stereochemistry outcome.

The combination of fluorination and the Robinson annulation as a one-pot reaction for fluorinated cyclohexenone **3a** was then attempted (Table 2). The fluorination step does not need to be asymmetric because both stereocenters were generated during the subsequential Michael addition. The fluorination reaction of β-ketoester **8** with SelectFluor™ was conducted under microwave heating at 120 °C for 20 min without using a catalyst. After the completion of the fluorination, chalcone **6a**, CF_3_C_6_H_4_CO_2_H and catalyst **cat-1** were added to the reaction mixture at 25 °C for the Robinson annulation. But this one-pot fluorination/Robinson annulation only gave **3a** in <10% yield probably due to the effect of the acidic SelectFluor™ derivative in the reaction mixture. To address this issue, bases including Na_2_CO_3_, K_2_CO_3_, and Cs_2_CO_3_ were used for the reaction. It was found that addition of 1.5 equiv. of Na_2_CO_3_ gave **3a** in 82% yield with a good ee and dr (Table 2, entry 6).

**Scheme 2:**
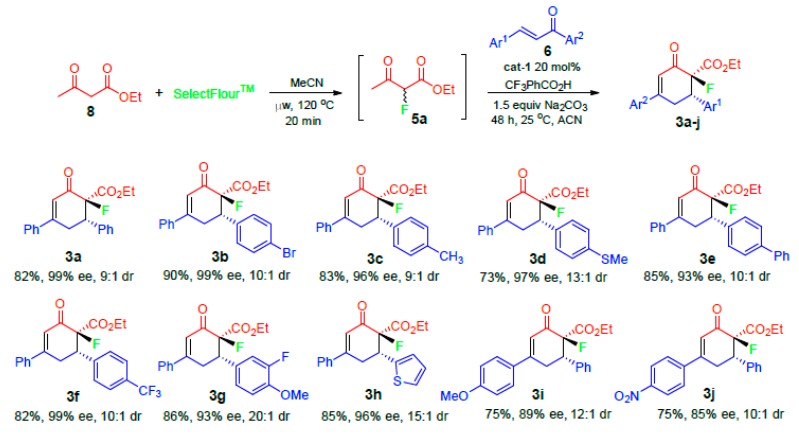
One-pot synthesis of 6-fluorocyclohex-2-en-1-ones **3**.

With the optimized conditions in hand, the scope of the one-pot synthesis of 6-fluorocyclohexenones **3a**–**j** using different Michael acceptor **6** was investigated (Scheme 2). Substrates **6** with electron-donating (SMe, Ph) and electron-withdrawing (Br, CF_3_) groups on Ar^1^ gave products **3b**–**f** in 82–90% yields with >96% ee and >9:1 dr. Reaction of a chalcone bearing a thiophene ring also gave product **3h** in a good yield and ee. The introduction of substituents on Ar^2^ of **6** gave products **3i** and **3j** with decreased ee, probably due to an unfavorable stereoelectronic effect.

**Scheme 3:**
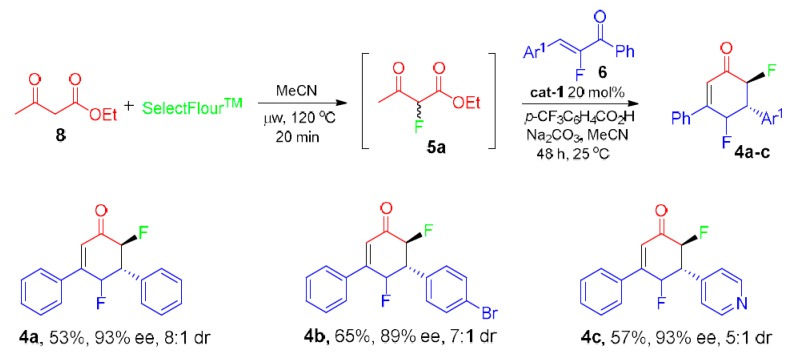
One-pot synthesis of 4,6-difluorocyclohexanones 4.

We have recently reported a one-pot Robinson annulation/dehydrofluorination/aromtization sequence for fluorinated phenols **2** using α-fluoro-β-ketoesters and α-fluoro-α,β-unsaturated ketones as substrates [24]. We envisioned that α-fluoro-α,β-unsaturated ketones **6** could be used for asymmetric synthesis of 4,6-difluorocyclohexanones **4** under organocatalytic conditions. Indeed, one-pot reactions of β-ketoester **8** went smoothly to afford **4a**–**c** in 53–65% yields with 5:1-8:1 dr and 89–93% ee (Scheme 3). However, all of these reactions afforded **4a**–**c** as decarboxylated products, even at low reaction temperatures (−30–0 °C). No aromatization products were observed under the reaction temperature without heating [24]. The products **4a**–**c** were found not to be stable during the workup and rotary vapor concentration of the crude product.

## 3. Conclusions

In summary, a one-pot fluorination and cinchona alkaloid amine-promoted organocatalytic Robinson sequence for asymmetric synthesis of fluorocyclohexenones bearing two stereocenters has been developed. Both stereocenters were established during the step of Michael addition to afford fluorinated cyclohexanones with up to 99% ee and 20:1 dr. Using fluorinated chalcones as Michael acceptors, 4,6-diflorocyclohexanones bearing three stereocenters were also synthesized stereoselectively and in good yields.

## Figures and Tables

**Table 1:**
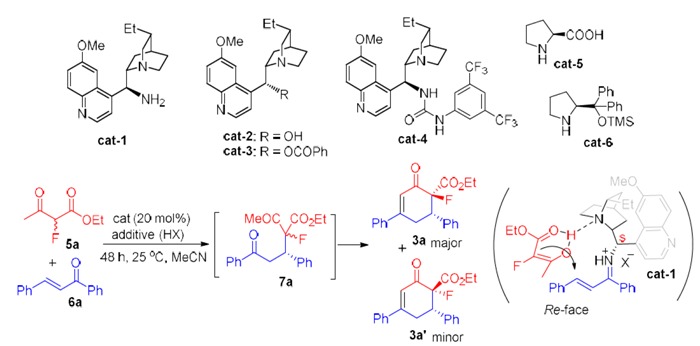
Screening of catalysts for the Robinson annulation ^a^.

Entry	Cat	Additive	Yield (%) ^b^	dr ^c^	ee (%) ^d^
1	**cat-1**	C_6_H_5_CO_2_H	79	5:1	99
2	**cat-2**	C_6_H_5_CO_2_H	NR	-	-
3	**cat-3**	C_6_H_5_CO_2_H	NR	-	-
4	**cat-4**	C_6_H_5_CO_2_H	NR	-	-
5	**cat-5**	C_6_H_5_CO_2_H	trace	-	-
6	**cat-6**	C_6_H_5_CO_2_H	27	3:1	85
7	**cat-1**	None	42	5:1	92
8	**cat-1**	AcOH	52	6:1	92
9	**cat-1**	TsOH	45	5:1	95
10	**cat-1**	CF_3_C_6_H_4_CO_2_H	89	9:1	99
11	**cat-1**	CCl_3_CO_2_H	75	8:1	90
12	**cat-1**	CF_3_CO_2_H	77	8:1	90
13^e^	**cat-1**	CF_3_C_6_H_4_CO_2_H	51	12:1	99

^a^ Reaction of 0.1 mmol of **5a** and 0.15 mmol of **6a** in 0.5 mL of MeCN. ^b^ Isolated yield. ^c^ Determined by ^1^H-NMR of crude reaction mixture. ^d^ Determined by chiral HPLC. ^e^ Reaction was carried out at −20 °C.

**Table 2:**
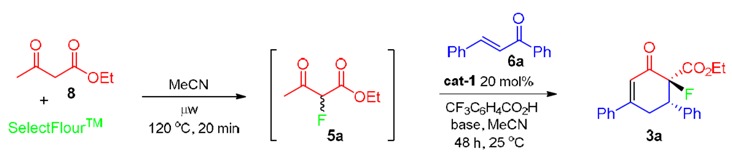
One-pot synthesis of 6-fluorocyclohex-2-en-1-ones 3 ^a^.

Entry	Base (equiv)	Yield (%) ^b^	dr ^c^	ee (%) ^d^
1	None	<10	-	-
2	Na_2_CO_3_ (0.5)	42	9:1	99
3	K_2_CO_3_ (0.5)	67	3:1	85
4	Cs_2_CO_3_(0.5)	85	1.5:1	52
5	Na_2_CO_3_ (1.0)	57	9:1	99
6	Na_2_CO_3_ (1.5)	82	9:1	99
7	Na_2_CO_3_ (2.0)	83	9:1	99

^a^ Reaction of 0.1 mmol of **8**, 0.15 mmol of SelectFluor™, 0.15 mmol of **6a** in 0.5 mL of MeCN. ^b^ Yield of isolated product **3a**. ^c^ Determined by ^1^H-NMR. ^d^ Determined by HPLC on Venusil Chiral OD-H column with 90:10 hexane/*i*-PrOH.

## Supplementary Materials

Supplementary Materials are available online, General procedure; characterization of ^1^H and ^13^C NMR spectra of products, HPLC spectra of racemic and enantioenriched products.
