# Supplementary material for: One-pot Fluorination and Organocatalytic Robinson Annulation for Asymmetric Synthesis of Mono- and Difluorinated Cyclohexenones
Source: Molecules. 2018 Sep 4;23(9):2251. doi: 10.3390/molecules23092251 (PMC6225330; doi:10.3390/molecules23092251)

One-pot Fluorination and Organocatalytic Robinson Annulation for Asymmetric Synthesis of Mono- and Difluorinated Cyclohexenones

Xin Huang [1,*] Weizhao Zhao [1], Xiaofeng Zhang [2], Miao Liu [2], Stanley N.S. Vasconcelos [3] and Wei Zhang [2,*]

1 College of Chemistry and Life Sciences, Zhejiang Normal University, Jinhua 321004, China; zhao792129582@163.com
2 Department of Chemistry, University of Massachusetts Boston, 100 Morrissey Boulevard, Boston, MA 02125, USA; Xiaofeng.Zhang002@umb.edu (X.Z.); liumiaomarcus@gmail.com (M.L.)
3 Departamento de Farmácia, Universidade de São Paulo, Av. Prof. Lineu Prestes, 580 São Paulo, SP 05508-000, Brasil; stanleynsv@gmail.com
* Correspondence: xin.huang@zjnu.cn (X.H.); wei2.zhang@umb.edu (W.Z.); Tel.: +1-617-287-6147 (W.Z.); Fax: +1-617-287-6030 (W.Z.)

Contents

1. General Information ……………………………………………………S2

2. Analytical Data of Products ………………………………………………S3

3. NMR Spectra of Products …………………………………………………S7

4. Chiral-LC of Products ……………………………………………………S27

1. General Information

Chemicals and solvents were purchased from commercial suppliers and used as received. ^{1}H and ^{13}C NMR spectra were recorded on a 400 MHz Agilent NMR spectrometer. Chemical shifts were reported in parts per million (ppm), and the residual solvent peak was used as an internal reference: proton (chloroform δ 7.26), carbon (chloroform δ 77.0). Multiplicity was indicated as follows: s (singlet), d (doublet), t (triplet), q (quartet), m (multiplet), dd (doublet of doublet), br s (broad singlet). Coupling constants were reported in Hertz (Hz). LC-MS were performed on an Agilent 2100 system. A C_{18} column (5.0 µm, 6.0 x 50 mm) was used for the separation. The mobile phases were methanol and water both containing 0.05% trifluoro acetic acid. A linear gradient was used to increase from 25:75 v/v methanol/water to 100% methanol over 7.0 min at a flow rate of 0.7 mL/min. UV detections were conducted at 210 nm, 254 nm and 365 nm. Low resolution mass spectra were recorded in APCI (atmospheric pressure chemical ionization). The high resolution mass spectra were obtained on a Waters Micromass GCT Premier. Sorbent silica gel XHL TLC plates (130815) were used for the thin-layer chromatography (TLC). Flash chromatography separations were performed on YAMAZEN AI-580 flash column system with Agela silica gel columns (230-400 µm mesh) and Angela Flash/Cheeta System with Venusil PrepG C_{18} column (10 µm, 120 Å, 21.2 mm x 250 mm). The enantiomeric excesses of products were determined by chiral phase HPLC analysis on a SHIMADZU LC-20AD system with Venusil Chiral CA, CD-H, and Regis (R,R)-Whelk-O1 column.

1.1 General procedure for Asymmetric Synthesis of Fluorinated Cyclohexanones:

To a solution of *β*-ketoesters **8** (0.1 mmol) in CH_3CN (0.5 mL) was added Selectfluor^{TM} (0.15 mmol). After being stirred and heated at 120 °C in microwave reaction station for 20 min, the reaction mixture was allowed to cool to room temperature and then **cat-1** (7 mg, 0.02 mmol), $CF_3C_6H_4COOH$ (4 mg, 0.02 mmol) and *α,β*-unsaturated ketones **6** (0.15 mmol) were added. The reaction mixture was stirred for 30 min before the addition of Na_2CO_3 (16 mg, 1.5 equiv.) After being stirred for 48 h, the resulting fluorinated cyclohexenones **3-4** were purified by YAMAZEN AI-580 flash column system with Agela silica gel columns (hexanes/ethyl acetate as eluent).

2. Analytical Data of Products

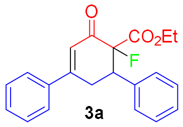

3a

The enantiomeric excess was determined by HPLC analysis (Venusil Chiral CD column, 90:10 hexane/i-PrOH, 1.0 mL/min, λ = 254 nm): tminor = 12.15 min, tmajor = 9.38 min, 99% ee.

^{1}H NMR (400 MHz, cdcl$_3$) δ 7.72 – 7.54 (m, 2H), 7.50 – 7.42 (m, 3H), 7.41 – 7.31 (m, 5H), 6.63 (dd, *J* = 2.9, 2.2 Hz, 1H), 4.15 (q, *J* = 7.1 Hz, 2H), 3.90 – 3.74 (m, 2H), 3.23 – 3.10 (m, 1H), 1.13 (t, *J* = 7.1 Hz, 3H).

^{19}F NMR (376 MHz, cdcl$_3$) δ -165.47.

^{13}C NMR (101 MHz, cdcl$_3$) δ 189.86 (d, *J* = 17.7 Hz), 165.61 (d, *J* = 26.3 Hz), 161.33, 137.42, 136.12, 130.94, 128.99, 128.69, 128.50, 128.36, 126.35, 122.61, 95.05 (d, *J* = 204.0 Hz), 62.3, 48.81 (d, *J* = 21.2 Hz), 32.64 (d, *J* = 4.0 Hz), 13.87.

HRMS (EI, m/z): calcd. for $C_{21}H_{19}FO_3$ $[M]^+$: 338.1318, Found: 338.1321.

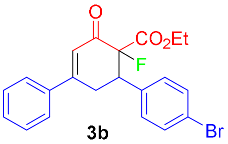

3b

The enantiomeric excess was determined by HPLC analysis (Venusil Chiral CD column, 95:5 hexane/*i*-PrOH, 1.0 mL/min, λ = 254 nm): tminor = 14.58 min, tmajor = 11.55 min, 99% ee.

^{1}H NMR (400 MHz, cdcl$_3$) δ 7.65 – 7.56 (m, 2H), 7.54 – 7.40 (m, 5H), 7.22 (d, *J* = 8.3 Hz, 2H), 6.62 (dd, *J* = 2.9, 2.1 Hz, 1H), 4.15 (q, *J* = 7.1 Hz, 2H), 3.88 – 3.71 (m, 2H), 3.20 – 3.06 (m, 1H), 1.16 (t, *J* = 7.1 Hz, 3H).

^{19}F NMR (376 MHz, cdcl$_3$) δ -165.24.

^{13}C NMR (101 MHz, cdcl$_3$) δ 189.42 (d, *J* = 17.2 Hz), 165.41 (d, *J* = 27.3 Hz), 161.01, 137.28, 135.08, 131.82, 131.02, 130.18, 129.01, 128.36, 126.32, 122.59, 94.72 (d, *J* = 204.0 Hz), 62.50, 48.21 (d, *J* = 21.2 Hz), 32.41 (d, *J* = 8.1 Hz), 13.90.

HRMS (EI, m/z): calcd. for $C_{21}H_{18}BrFO_3$ $[M]^+$: 416.0423, Found: 416.0426.

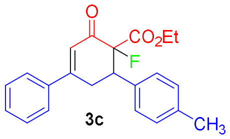

3c

The enantiomeric excess was determined by HPLC analysis (Venusil Chiral CD column, 95:5 hexane/*i*-PrOH, 1.0 mL/min, λ = 254 nm): tminor = 15.34 min, tmajor = 16.24 min, 96% ee.

^{1}H NMR (400 MHz, cdcl$_3$) δ 7.62 – 7.56 (m, 2H), 7.44 (qdd, *J* = 5.6, 3.6, 1.6 Hz, 3H), 7.30 (dd, *J* = 8.1, 1.2 Hz, 2H), 7.15 (dd, *J* = 8.3, 0.6 Hz, 2H), 6.61 (d, *J* = 2.0 Hz, 1H), 4.16 (q, *J* = 7.1, 2H), 4.00 – 3.80 (m, 1H), 3.35 (ddd, *J* = 18.3, 10.9, 2.4 Hz, 1H), 3.04 (dd, *J* = 18.3, 4.7 Hz, 1H), 2.34 (s, 3H), 1.13 (t, *J* = 7.1 Hz, 3H).

^{19}F NMR (376 MHz, cdcl$_3$) δ -175.16.

^{13}C NMR (101 MHz, cdcl$_3$) δ 188.58 (d, *J* = 17.2 Hz), 166.56 (d, J = 26.2 Hz), 161.24, 137.94, 137.43, 133.84, 130.92, 129.40, 128.97, 128.55 (d, J = 2.5 Hz), 126.30, 121.99, 94.59 (d, J = 197.2 Hz), 61.90, 47.24 (d, J = 22.0 Hz), 32.00 (d, J = 5.6 Hz), 21.09, 13.96.

HRMS (EI, m/z): calcd. for $C_{22}H_{21}FO_3$ $[M]^+$: 352.1475, Found: 352.1477.

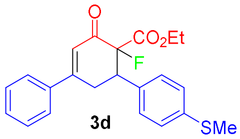

3d

The enantiomeric excess was determined by HPLC analysis (Venusil Chiral CD column, 95:5 hexane/*i*-PrOH, 1.0 mL/min, λ = 254 nm): tminor = 9.05 min, tmajor = 7.11 min, 97% ee.

^{1}H NMR (400 MHz, cdcl$_3$) δ 7.65 – 7.58 (m, 2H), 7.50 – 7.40 (m, 3H), 7.25 (s, 4H), 6.62 (dd, *J* = 2.9, 2.1 Hz, 1H), 4.16 (q, *J* = 7.2 Hz, 2H), 3.93 – 3.65 (m, 2H), 3.14 (ddt, *J* = 16.7, 13.8, 7.1 Hz, 1H), 2.49 (s, 3H), 1.15 (t, *J* = 7.1 Hz, 3H).

^{19}F NMR (376 MHz, cdcl$_3$) δ -165.53.

^{13}C NMR (101 MHz, cdcl$_3$) δ 188.76 (d, J = 18.2 Hz), 166.54 (d, J = 26.3 Hz), 161.24, 138.87, 137.40, 132.76, 130.94, 128.98, 128.90, 126.58, 126.27, 122.60, 95.01 (d, J = 204.0 Hz), 62.39, 48.32 (d, J = 22.2 Hz), 32.63 (d, J = 8.1 Hz), 15.65, 13.91.
HRMS (EI, m/z): calcd. for $C_{22}H_{21}FO_3S$ $[M]^+$: 384.1195, Found: 384.1196.

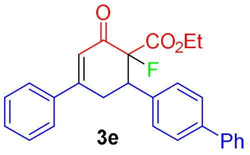

3e

The enantiomeric excess was determined by HPLC analysis (Venusil Chiral CA column, 90:10 hexane/*i*-PrOH, 1.0 mL/min, λ = 254 nm): tminor = 15.57 min, tmajor = 10.60 min, 93% ee.
^{1}H NMR (400 MHz, cdcl$_3$) δ 7.67 – 7.55 (m, 6H), 7.52 – 7.40 (m, 7H), 7.36 (dt, *J* = 9.4, 4.3 Hz, 1H), 6.64 (d, *J* = 2.1 Hz, 1H), 4.18 (q, *J* = 7.1, 2H), 4.00 (ddd, *J* = 35.8, 10.9, 4.7 Hz, 1H), 3.41 (ddd, *J* = 18.3, 11.0, 2.4 Hz, 1H), 3.10 (dd, *J* = 18.3, 4.7 Hz, 1H), 1.13 (t, *J* = 7.1 Hz, 3H).
^{19}F NMR (376 MHz, cdcl$_3$) δ -175.02.
^{13}C NMR (101 MHz, cdcl$_3$) δ 188.42 (d, J = 20.2 Hz), 166.7, 161.13, 140.45, 137.37, 130.98, 129.14, 129.12, 129.00, 128.80, 127.47, 127.41, 127.06, 127.03, 126.32, 122.02, 94.49 (d, J = 197.0 Hz), 62.02, 47.30 (d, J = 22.2 Hz), 31.92 (d, J = 6.1 Hz), 13.91.
HRMS (EI, m/z): calcd. for $C_{27}H_{23}FO_3$ $[M]^+$: 414.1631, Found: 414.1633.

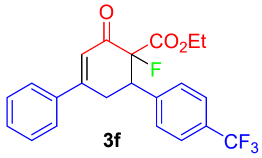

3f

The enantiomeric excess was determined by HPLC analysis (Venusil Chiral CA column, 90:10 hexane/*i*-PrOH, 1.0 mL/min, λ = 254 nm): tminor = 19.43 min, tmajor = 13.77 min, 99% ee.
^{1}H NMR (400 MHz, cdcl$_3$) δ 7.67 – 7.52 (m, 6H), 7.53 – 7.39 (m, 3H), 6.63 (d, *J* = 2.1 Hz, 1H), 4.17 (q, *J* = 7.1, 2H), 4.11 – 3.96 (m, 1H), 3.37 (ddd, *J* = 18.2, 11.0, 2.4 Hz, 1H), 3.07 (dd, *J* = 18.2, 4.8 Hz, 1H), 1.11 (t, *J* = 7.1 Hz, 3H).
^{19}F NMR (376 MHz, cdcl$_3$) δ -62.76, -174.96.
^{13}C NMR (101 MHz, cdcl$_3$) δ 187.82 (d, J = 20.2 Hz), 166.29 (d, J = 26.3 Hz), 160.67, 140.85, 137.12, 131.13, 130.36, 129.15 (d, J = 3.0 Hz), 129.06, 126.29, 125.71 (q, J = 3.7 Hz), 122.54, 94.00 (d, J = 198.0 Hz), 62.19, 47.31 (d, J = 22.2 Hz), 31.66 (d, J = 6.1 Hz), 13.91.
HRMS (EI, m/z): calcd. for $C_{22}H_{18}F_4O_3$ $[M]^+$: 406.1192, Found: 406.1192.

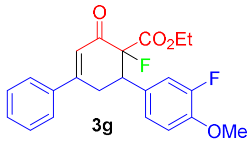

3g

The enantiomeric excess was determined by HPLC analysis (Venusil Chiral CD column, 95:5 hexane/*i*-PrOH, 1.0 mL/min, λ = 254 nm): tminor = 3.25 min, tmajor = 3.73 min, 93% ee.
^{1}H NMR (400 MHz, cdcl$_3$) δ 7.63 – 7.55 (m, 2H), 7.51 – 7.39 (m, 3H), 7.10 – 6.99 (m, 2H), 6.95 – 6.90 (m, 1H), 6.61 (d, *J* = 2.0 Hz, 1H), 4.18 (q, *J* = 7.2 Hz, 2H), 4.02 – 3.81 (m, 4H), 3.32 (ddd, *J* = 18.2, 11.0, 2.4 Hz, 1H), 3.06 (dd, *J* = 18.2, 4.7 Hz, 1H), 1.14 (t, *J* = 7.1 Hz, 3H).
^{19}F NMR (376 MHz, cdcl$_3$) δ -135.60, -175.19.
^{13}C NMR (101 MHz, cdcl$_3$) δ 188.20 (d, J = 20.2 Hz), 166.47 (d, J = 27.3 Hz), 160.93 (s), 152.2 (d, J = 248.5 Hz), 147.63 (d, J = 10.8 Hz), 137.24, 133.30 (d, J = 3.6 Hz), 132.82, 131.05, 129.02, 128.55 (d, J = 16.6 Hz), 126.31, 121.98, 121.22 (d, J = 7.0 Hz), 116.13 (d, J = 18.4 Hz), 113.86, 94.40 (d, J = 198.0 Hz), 62.05, 56.30, 47.19, 3(d, J = 22.2 Hz), 2.09 (d, J = 5.4 Hz), 14.01.
HRMS (EI, m/z): calcd. for $C_{22}H_{20}F_2O_3$ $[M]^+$: 386.1330, Found: 386.1342.

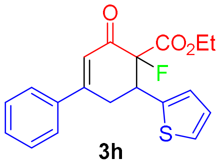

3h

The enantiomeric excess was determined by HPLC analysis (Venusil Chiral CD column, 95:5 hexane/*i*-PrOH, 1.0 mL/min, λ = 254 nm): tminor = 18.64 min, tmajor = 14.40 min, 96% ee.

^{1}H NMR (400 MHz, cdcl$_3$) δ 7.68 – 7.56 (m, 2H), 7.52 – 7.41 (m, 3H), 7.28 (dd, *J* = 5.1, 1.2 Hz, 1H), 7.10 (ddd, *J* = 3.5, 2.0, 0.9 Hz, 1H), 7.03 (dd, *J* = 5.1, 3.6 Hz, 1H), 6.66 – 6.58 (m, 1H), 4.25 – 4.06 (m, 3H), 3.75 (ddd, *J* = 18.0, 11.7, 2.5 Hz, 1H), 3.32 (ddd, *J* = 18.0, 5.2, 2.4 Hz, 1H), 1.15 (t, *J* = 7.1 Hz, 3H).

^{19}F NMR (376 MHz, cdcl$_3$) δ -163.76.

^{13}C NMR (101 MHz, cdcl$_3$) δ 189.28 (d, J = 17.7 Hz), 165.24, 160.58, 138.63, 137.23, 131.01, 129.02, 127.01, 126.76, 126.34, 125.10, 122.67, 94.63 (d, J = 203.0 Hz), 62.50, 44.10 (d, J = 22.2 Hz), 34.02 (d, J = 7.7 Hz), 13.83.

HRMS (EI, m/z): calcd. for $C_{19}H_{17}FO_3S$ $[M]^+$: 344.0882, Found: 344.0990.

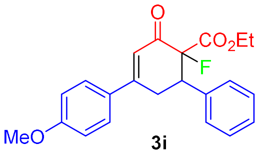

3i

The enantiomeric excess was determined by HPLC analysis (Venusil Chiral CD column, 95:5 hexane/*i*-PrOH, 1.0 mL/min, λ = 254 nm): tminor = 24.75 min, tmajor = 12.14 min, 89% ee.

^{1}H NMR (400 MHz, cdcl$_3$) δ 7.63 – 7.53 (m, 2H), 7.41 (dt, *J* = 8.0, 1.4 Hz, 2H), 7.39 – 7.28 (m, 3H), 7.01 – 6.89 (m, 2H), 6.58 (d, *J* = 2.0 Hz, 1H), 4.14 (dq, *J* = 7.1, 3.5 Hz, 2H), 4.02 – 3.79 (m, 4H), 3.32 (ddd, *J* = 18.1, 11.0, 2.3 Hz, 1H), 3.06 (dd, *J* = 18.1, 4.8 Hz, 1H), 1.09 (t, *J* = 7.1 Hz, 3H).

^{19}F NMR (376 MHz, cdcl$_3$) δ -174.75.

^{13}C NMR (101 MHz, cdcl$_3$) δ 188.38 (d, J = 20.2 Hz), 166.72 (d, J = 25.4 Hz), 162.06, 160.42, 137.02, 129.33, 128.75, 128.72, 128.17, 128.03, 120.00, 114.39, 94.51 (d, J = 196.0 Hz), 61.87, 55.45, 47.54(d, J = 22.2 Hz), 31.57 (d, J = 5.4 Hz), 13.93.

HRMS (EI, m/z): calcd. for $C_{22}H_{21}FO_4$ $[M]^+$: 368.1424, Found: 368.1421.

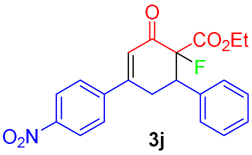

3j

The enantiomeric excess was determined by HPLC analysis (Venusil Chiral CD column, 95:5 hexane/*i*-PrOH, 1.0 mL/min, λ = 254 nm): tminor = 15.74 min, tmajor = 14.36 min,85% ee.

^{1}H NMR (400 MHz, cdcl$_3$) δ 8.28 – 8.16 (m, 2H), 7.67 – 7.55 (m, 4H), 7.53 – 7.39 (m, 3H), 6.64 (d, *J* = 2.0 Hz, 1H), 4.23 – 4.02 (m, 3H), 3.37 (ddd, *J* = 18.1, 11.0, 2.4 Hz, 1H), 3.08 (dd, *J* = 18.1, 4.8 Hz, 1H), 1.14 (t, *J* = 7.1 Hz, 3H).

^{19}F NMR (376 MHz, cdcl$_3$) δ -174.84.

^{13}C NMR (101 MHz, cdcl$_3$) δ 187.43 (d, J = 20.2 Hz), 166.12 (d, J = 25.3 Hz), 160.40, 147.79, 144.06, 136.96, 131.25, 129.77 (d, J = 2.9 Hz), 129.11, 126.29, 123.95, 122.02, 93.73 (d, J = 197.0 Hz), 62.36, 47.19 (d, J = 21.2 Hz), 31.49 (d, J = 5.2 Hz), 14.00.

HRMS (EI, m/z): calcd. for $C_{21}H_{18}FNO_5$ $[M]^+$: 383.1169, Found383.1167.

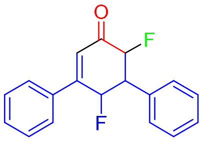

4a

The enantiomeric excess was determined by HPLC analysis (Venusil Chiral CD column, 90:10 hexane/*i*-PrOH, 1.0 mL/min, λ = 254 nm): tminor = 16.54 min, tmajor = 14.48 min, 93% ee.

^{1}H NMR (400 MHz, cdcl$_3$) δ 7.63 – 7.55 (m, 2H), 7.52 (ddd, *J* = 4.0, 3.4, 1.6 Hz, 2H), 7.49 – 7.41 (m, 2H), 7.34 – 7.28 (m, 2H), 7.27 – 7.24 (m, 2H), 6.48 (d, *J* = 4.3 Hz, 1H), 5.92 (ddd, *J* = 11.4, 9.4, 1.5 Hz, 1H), 5.22 (ddd, *J* = 47.6, 12.8, 0.8 Hz, 1H), 3.94 – 3.70 (m, 1H).

^{19}F NMR (376 MHz, cdcl$_3$) δ -177.97, -196.93.
^{13}C NMR (101 MHz, cdcl$_3$) δ 202.07, 191.22, 156.95, 133.80, 132.66, 132.26, 130.98, 129.79, 128.93, 127.37, 127.35, 124.44, 122.51, 90.70, 89.99, 79.02, 77.30, 77.18, 76.98, 76.66, 63.70, 53.41.
HRMS (ESI, m/z): calcd. for $C_{18}H_{14}F_2ONa$ $[M+Na]^+$: 307.0910, Found: 307.0910.

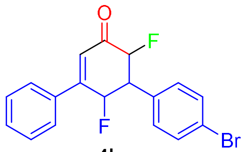

4b

The enantiomeric excess was determined by HPLC analysis (Venusil Chiral CD column, 90:10 hexane/*i*-PrOH, 1.0 mL/min, λ = 254 nm): tminor = 12.53 min, tmajor = 9.39 min, 89% ee.
^{1}H NMR (400 MHz, cdcl$_3$) δ 7.58 – 7.49 (m, 2H), 7.50 – 7.32 (m, 7H), 6.48 (d, *J* = 4.3 Hz, 1H), 5.97 (ddd, *J* = 46.8, 9.3, 1.6 Hz, 1H), 5.26 (ddd, *J* = 47.6, 12.8, 0.8 Hz, 1H), 3.98 – 3.68 (m, 1H).
^{19}F NMR (376 MHz, cdcl$_3$) δ -177.60, -196.92.
^{13}C NMR (101 MHz, cdcl$_3$) δ 191.92 (d, J = 15.7 Hz), 157.37 (d, J = 21.2 Hz), 134.83, 133.93, 130.87, 129.08, 128.89, 128.70, 128.44, 128.11, 127.38 (d, J = 1.9 Hz), 124.47 (d, J = 4.6 Hz), 90.64 (d, J = 11.4 Hz), 90.30 (dd, J = 34.3, 10.3 Hz), 88.60 (dd, J = 20.4, 10.9 Hz), 54.14 (m), 25.35.
HRMS (EI, m/z): calcd. for $C_{18}H_{13}BrF_2O$ $[M]^+$: 362.0118, Found: 362.0127.

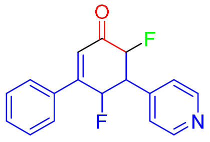

4c

The enantiomeric excess was determined by HPLC analysis (Venusil Chiral CD column, 90:10 hexane/*i*-PrOH, 1.0 mL/min, λ = 254 nm): tminor = 28.12 min, tmajor = 20.48 min, 93% ee.
^{1}H NMR (400 MHz, cdcl$_3$) δ 8.71 (d, *J* = 5.8 Hz, 2H), 7.52 (ddt, *J* = 6.1, 4.3, 2.0 Hz, 2H), 7.50 – 7.42 (m, 2H), 7.37 (d, *J* = 6.0 Hz, 2H), 7.27 – 7.25 (m, 1H), 6.50 (d, *J* = 4.4 Hz, 1H), 5.97 (ddd, *J* = 47.0, 9.4, 1.6 Hz, 1H), 5.26 (ddd, *J* = 47.7, 12.9, 0.8 Hz, 1H), 3.99 – 3.67 (m, 1H).
^{19}F NMR (376 MHz, cdcl$_3$) δ -177.98, -196.91.
^{13}C NMR (101 MHz, cdcl$_3$) δ 176.46, 150.50, 143.73, 131.11, 128.99, 127.36, 127.34, 124.45, 123.35, 89.49, 87.70, 77.30, 76.98, 76.66, 53.19, 39.15, 38.29.
HRMS (EI, m/z): calcd. for $C_{17}H_{13}F_2NO$ $[M]^+$: 285.0965, Found: 285.0963.

3. NMR Spectra of Products

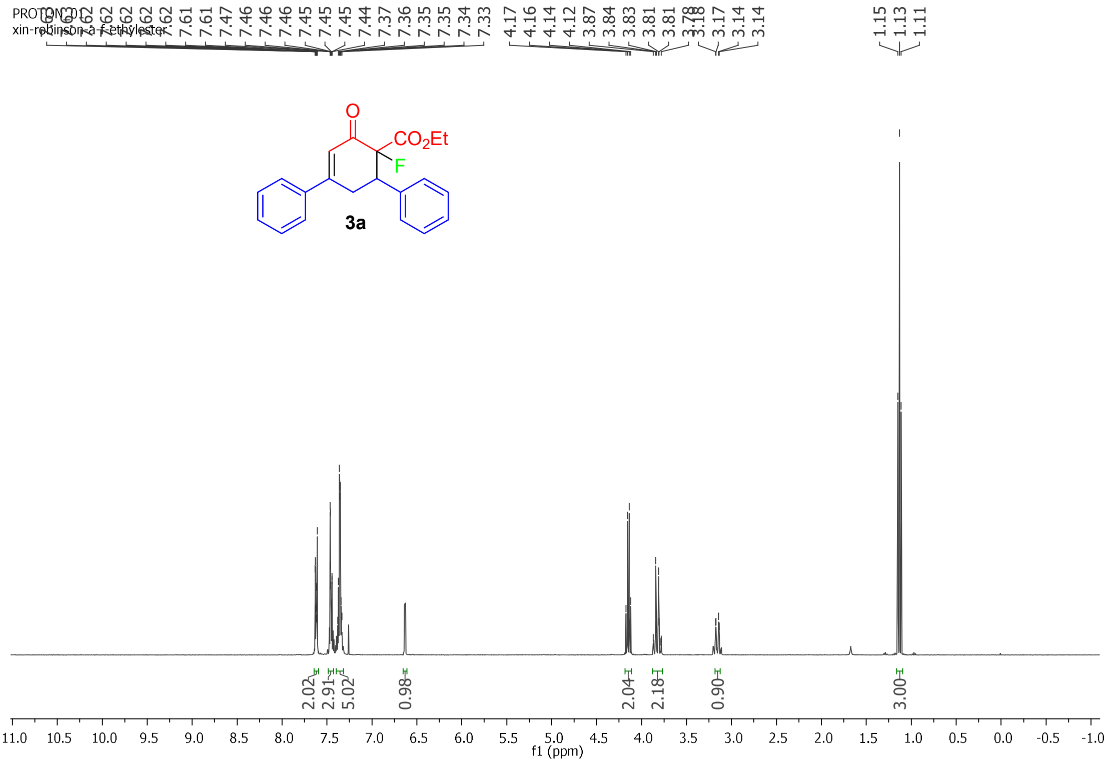

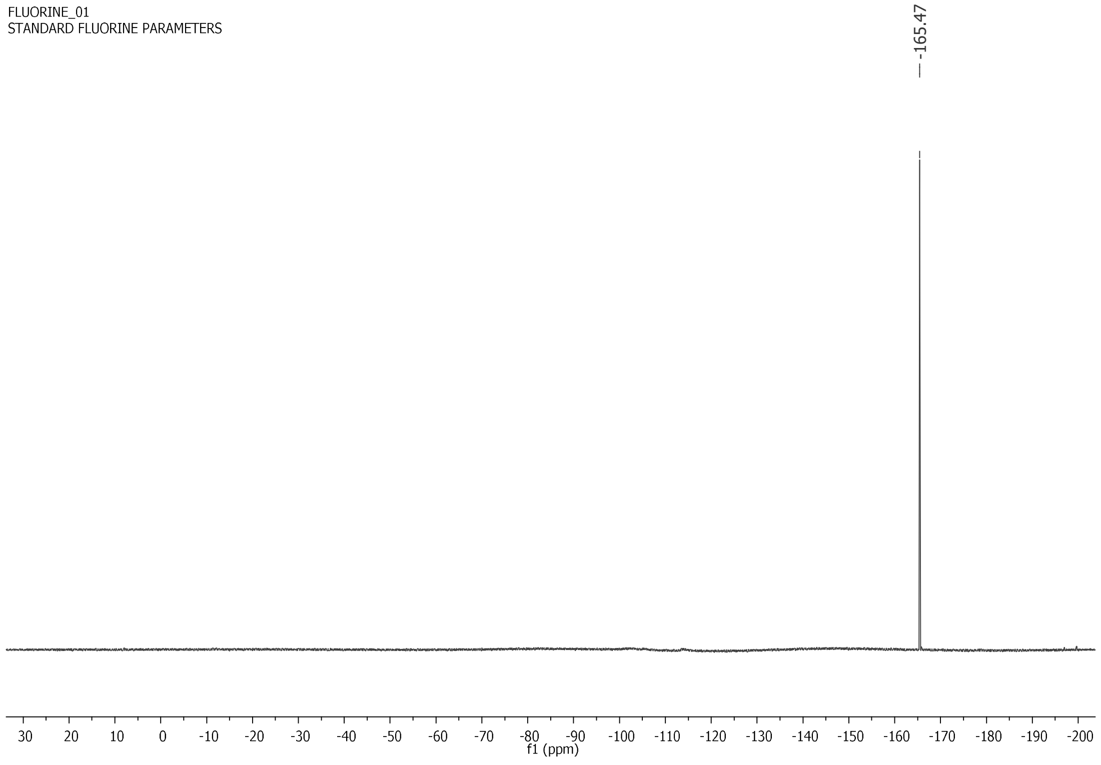

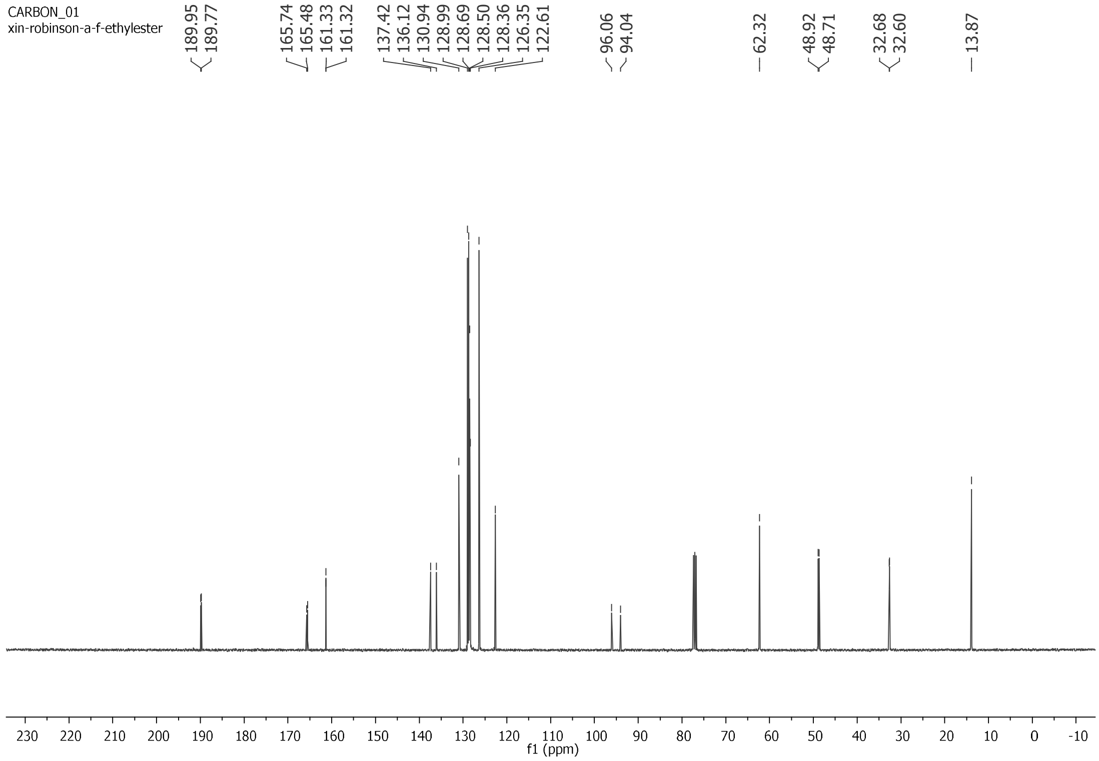
CARBON_01
xin-robinson-a-f-ethylester
189.95
189.77
165.74
165.48
161.33
161.32
137.42
136.12
130.94
128.99
128.69
128.50
128.36
126.35
122.61
96.06
94.04
62.32
48.92
48.71
32.68
32.60
13.87
230 220 210 200 190 180 170 160 150 140 130 120 110 100 90 80 70 60 50 40 30 20 10 0 -10
f1 (ppm)

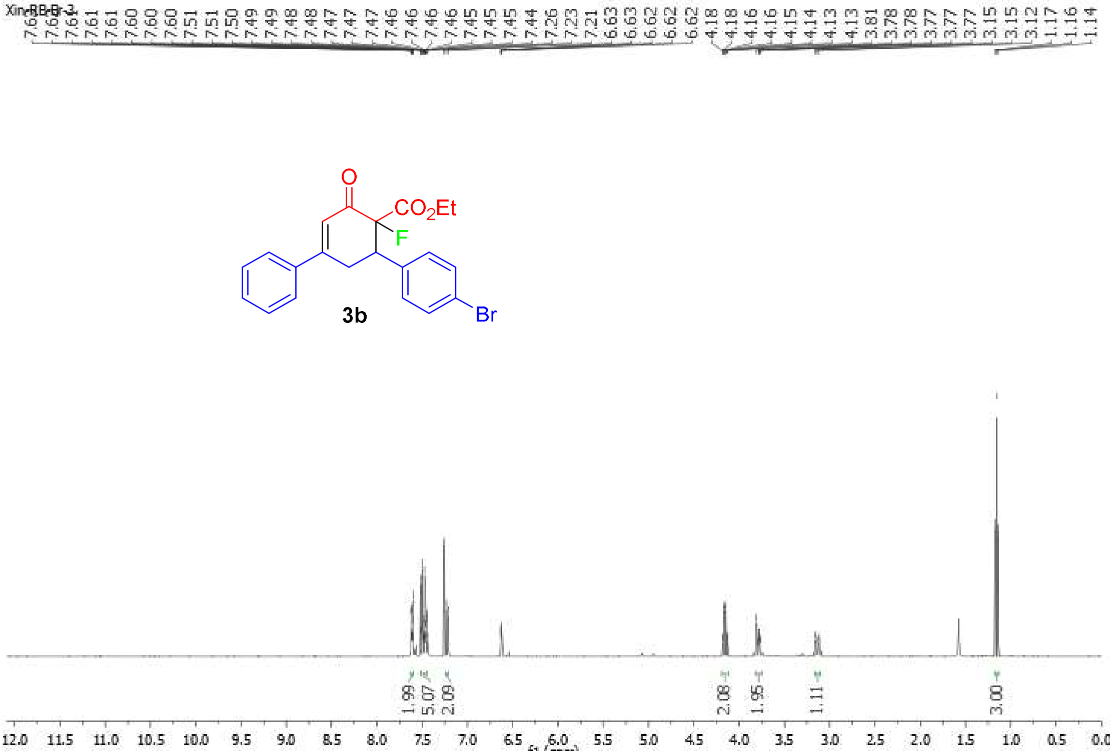
Xin-RB-Br-3
7.63 7.62 7.61 7.61 7.60 7.60 7.60 7.51 7.51 7.50 7.49 7.49 7.48 7.48 7.47 7.47 7.47 7.46 7.46 7.46 7.45 7.45 7.45 7.44 7.26 7.23 7.21 6.63 6.63 6.62 6.62 4.18 4.18 4.16 4.16 4.15 4.14 4.13 4.13 3.81 3.78 3.78 3.77 3.77 3.77 3.15 3.15 3.12 1.17 1.16 1.14
O
CO2Et
F
Br
3b
1.99
5.07
2.09
2.08
1.95
1.11
3.00
12.0 11.5 11.0 10.5 10.0 9.5 9.0 8.5 8.0 7.5 7.0 6.5 6.0 5.5 5.0 4.5 4.0 3.5 3.0 2.5 2.0 1.5 1.0 0.5 0.0

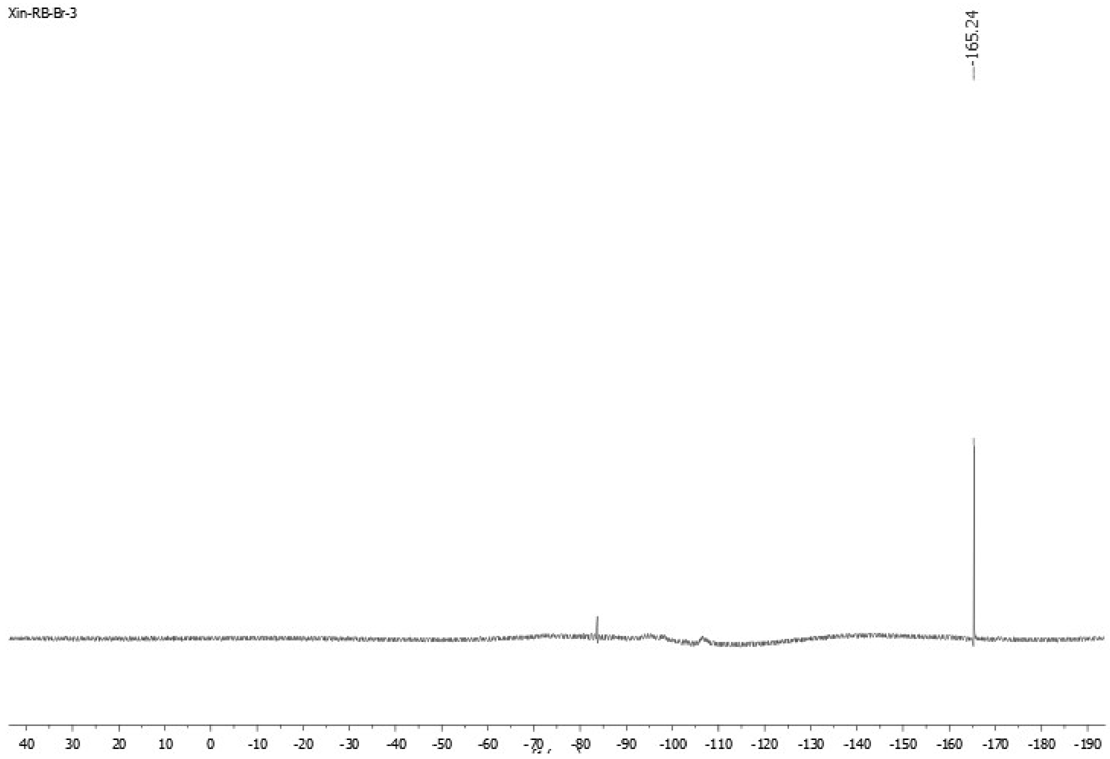
Xin-RB-Br-3
-165.24
40 30 20 10 0 -10 -20 -30 -40 -50 -60 -70 -80 -90 -100 -110 -120 -130 -140 -150 -160 -170 -180 -190

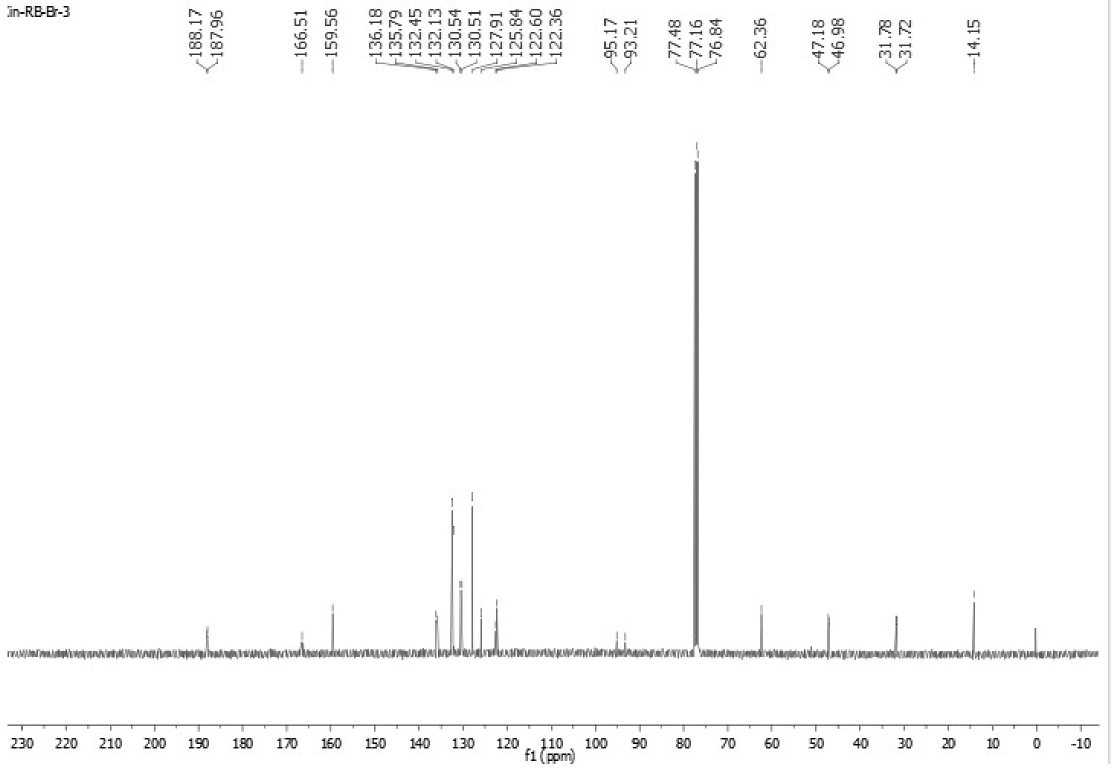
Xin-RB-Br-3
188.17
187.96
166.51
159.56
136.18
135.79
132.45
132.13
130.54
130.51
127.91
125.84
122.60
122.36
95.17
93.21
77.48
77.16
76.84
62.36
47.18
46.98
31.78
31.72
14.15
230 220 210 200 190 180 170 160 150 140 130 120 110 100 90 80 70 60 50 40 30 20 10 0 -10
f1 (ppm)

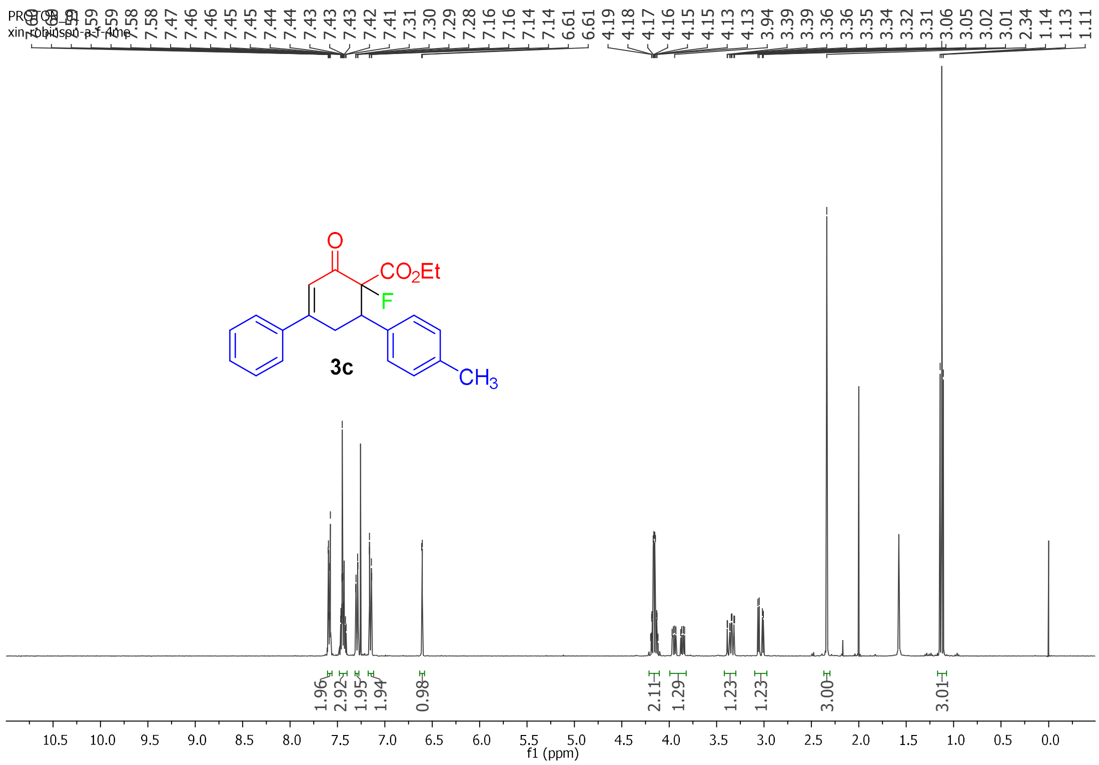
PROTON_01
xin-robinson-a-f-4me
7.59 7.58 7.47 7.46 7.45 7.45 7.44 7.44 7.43 7.43 7.43 7.42 7.41 7.31 7.30 7.29 7.28 7.16 7.16 7.14 7.14 6.61 6.61
4.19 4.18 4.17 4.16 4.15 4.15 4.13 4.13 3.94 3.39 3.39 3.36 3.36 3.35 3.34 3.32 3.31 3.06 3.05 3.02 3.01 2.34 1.14 1.13 1.11
O
CO2Et
F
CH3
3c
1.96 2.92 1.95 1.94 0.98 2.11 1.29 1.23 1.23 3.00 3.01
10.5 10.0 9.5 9.0 8.5 8.0 7.5 7.0 6.5 6.0 5.5 5.0 4.5 4.0 3.5 3.0 2.5 2.0 1.5 1.0 0.5 0.0
f1 (ppm)

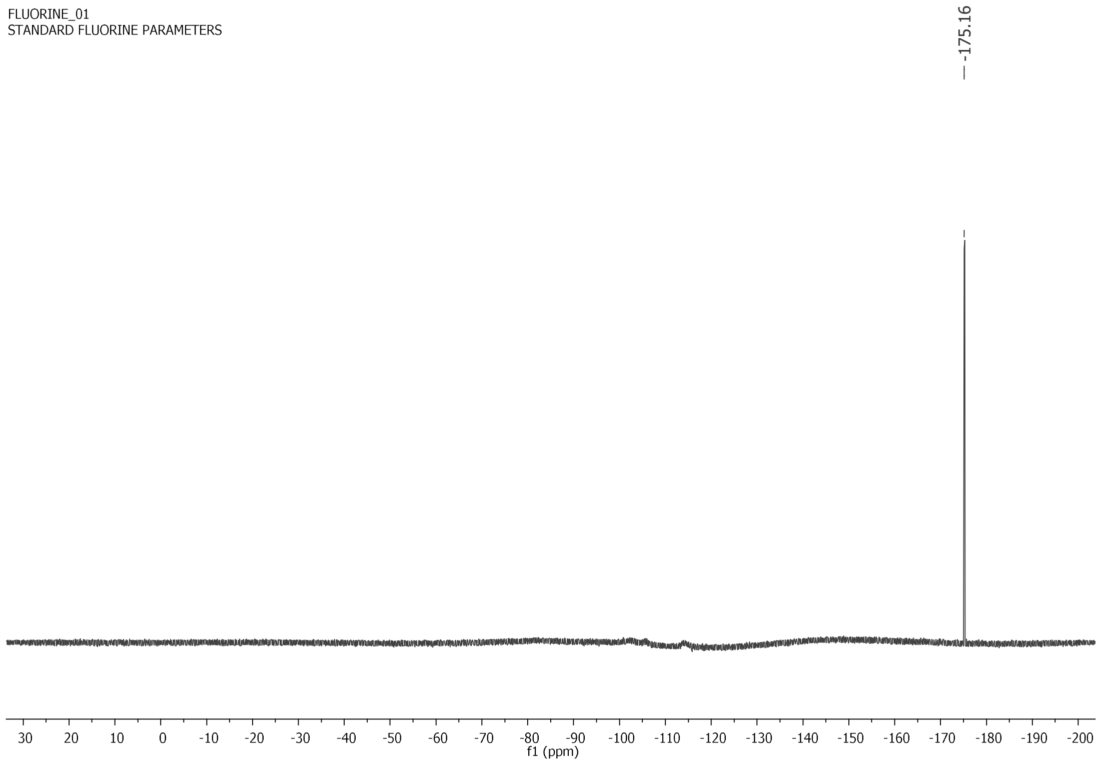
FLUORINE_01
STANDARD FLUORINE PARAMETERS
-175.16
30 20 10 0 -10 -20 -30 -40 -50 -60 -70 -80 -90 -100 -110 -120 -130 -140 -150 -160 -170 -180 -190 -200
f1 (ppm)

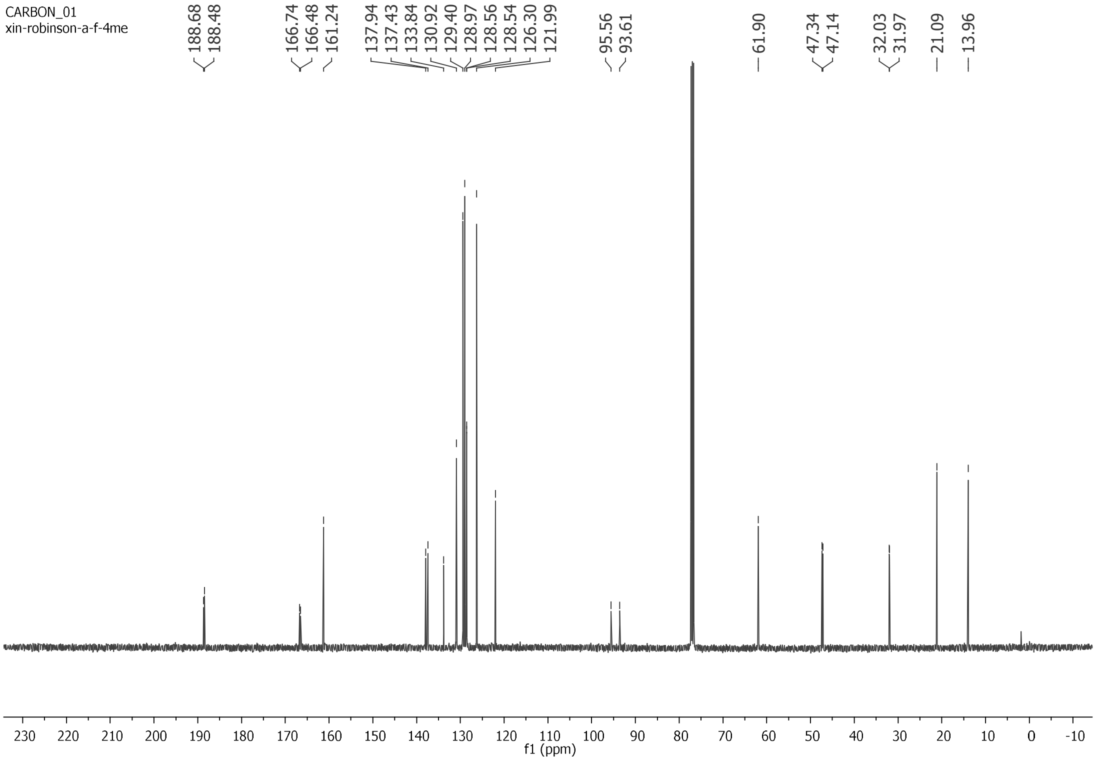
CARBON_01
xin-robinson-a-f-4me
188.68
188.48
166.74
166.48
161.24
137.94
137.43
133.84
130.92
129.40
128.97
128.56
128.54
126.30
121.99
95.56
93.61
61.90
47.34
47.14
32.03
31.97
21.09
13.96
230 220 210 200 190 180 170 160 150 140 130 120 110 100 90 80 70 60 50 40 30 20 10 0 -10
f1 (ppm)

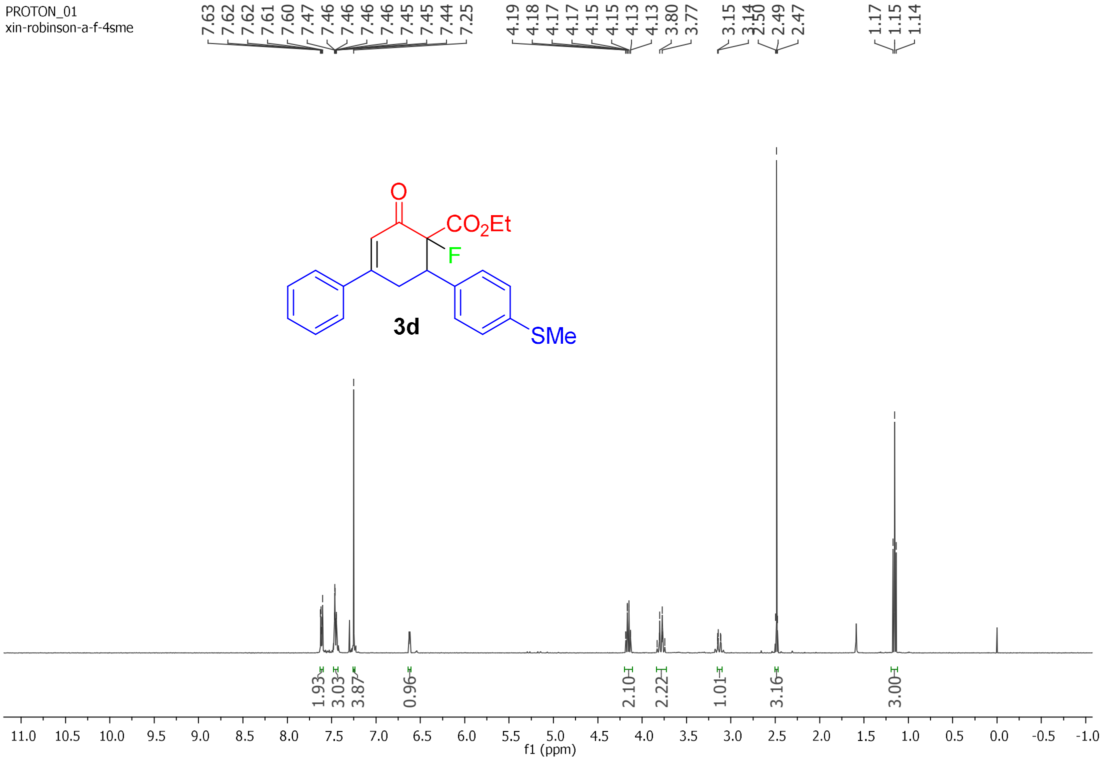
PROTON_01
xin-robinson-a-f-4sme
7.63
7.62
7.62
7.61
7.60
7.47
7.46
7.46
7.46
7.46
7.45
7.45
7.44
7.25
4.19
4.18
4.17
4.17
4.15
4.15
4.13
4.13
3.80
3.77
3.15
3.14
2.50
2.49
2.47
1.17
1.15
1.14
O
CO_2Et
F
3d
SMe
1.93
3.03
3.87
0.96
2.10
2.22
1.01
3.16
3.00
11.0 10.5 10.0 9.5 9.0 8.5 8.0 7.5 7.0 6.5 6.0 5.5 5.0 4.5 4.0 3.5 3.0 2.5 2.0 1.5 1.0 0.5 0.0 -0.5 -1.0
f1 (ppm)

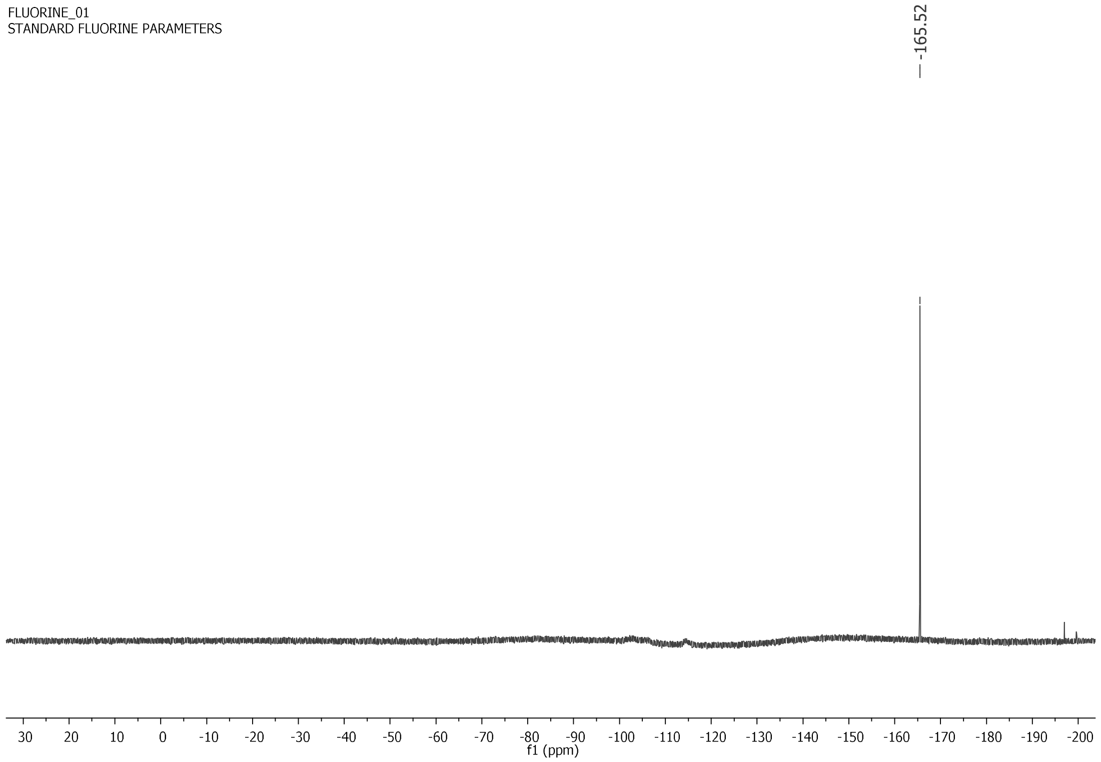
FLUORINE_01
STANDARD FLUORINE PARAMETERS
-165.52
30 20 10 0 -10 -20 -30 -40 -50 -60 -70 -80 -90 -100 -110 -120 -130 -140 -150 -160 -170 -180 -190 -200
f1 (ppm)

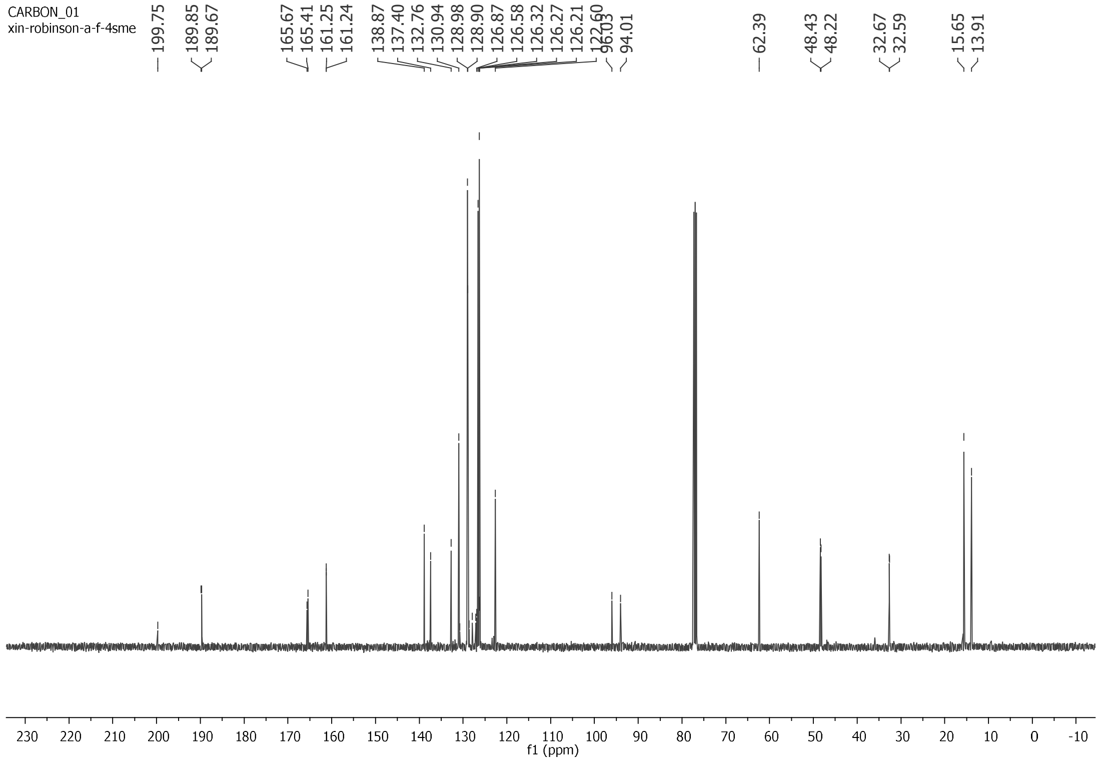
CARBON_01
xin-robinson-a-f-4sme
199.75
189.85
189.67
165.67
165.41
161.25
161.24
138.87
137.40
132.76
130.94
128.98
128.90
126.87
126.58
126.32
126.27
126.21
122.60
96.03
94.01
62.39
48.43
48.22
32.67
32.59
15.65
13.91
230 220 210 200 190 180 170 160 150 140 130 120 110 100 90 80 70 60 50 40 30 20 10 0 -10
f1 (ppm)

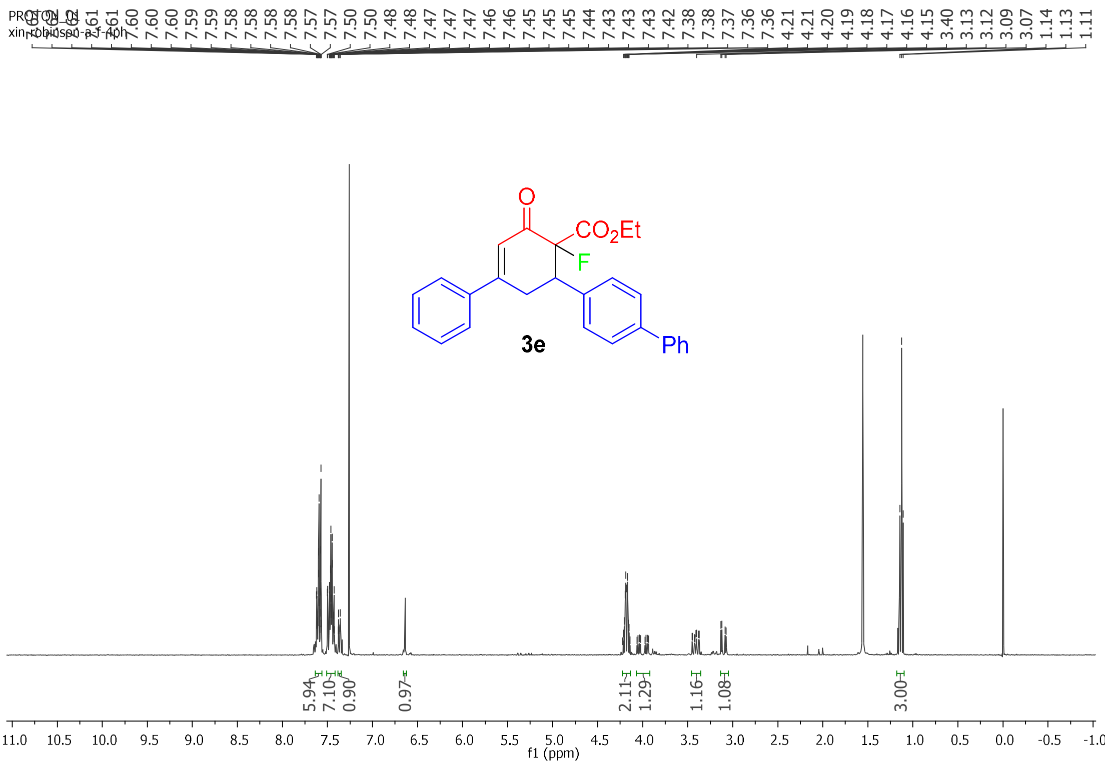
PROTON_01
xin-robinson-a-f-4ph
O
CO2Et
F
3e
Ph
5.94
7.10
0.90
0.97
2.11
1.29
1.16
1.08
3.00
f1 (ppm)

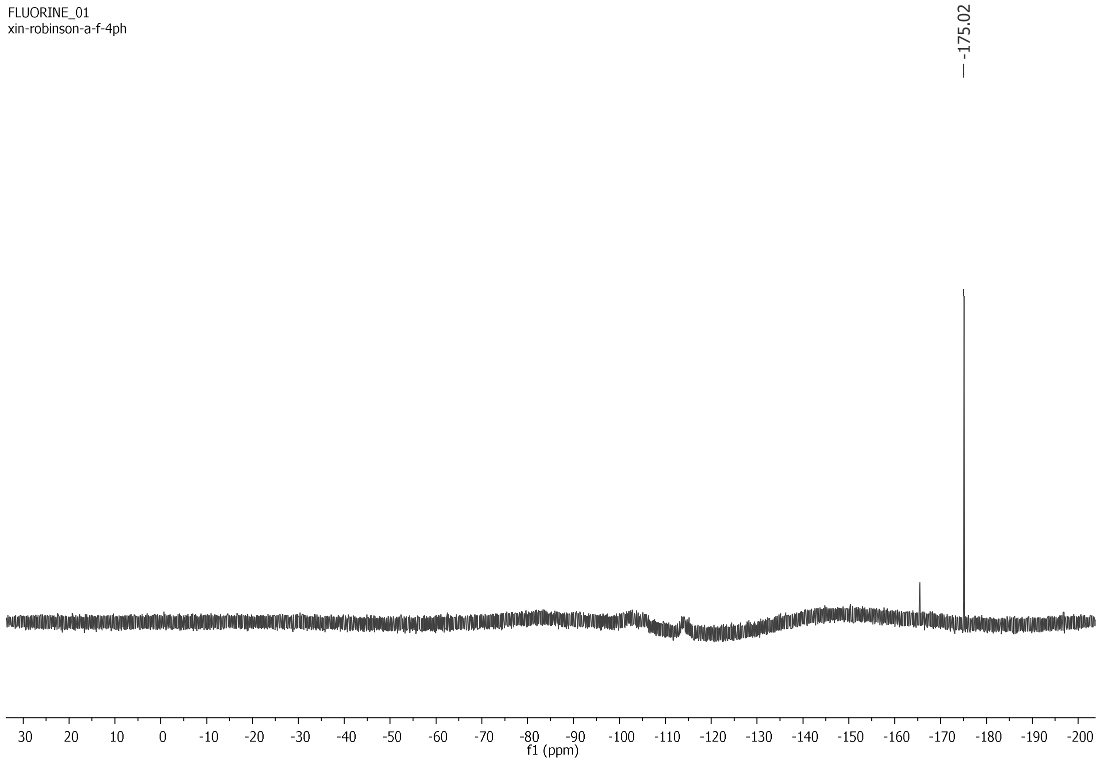
FLUORINE_01
xin-robinson-a-f-4ph
-175.02
f1 (ppm)

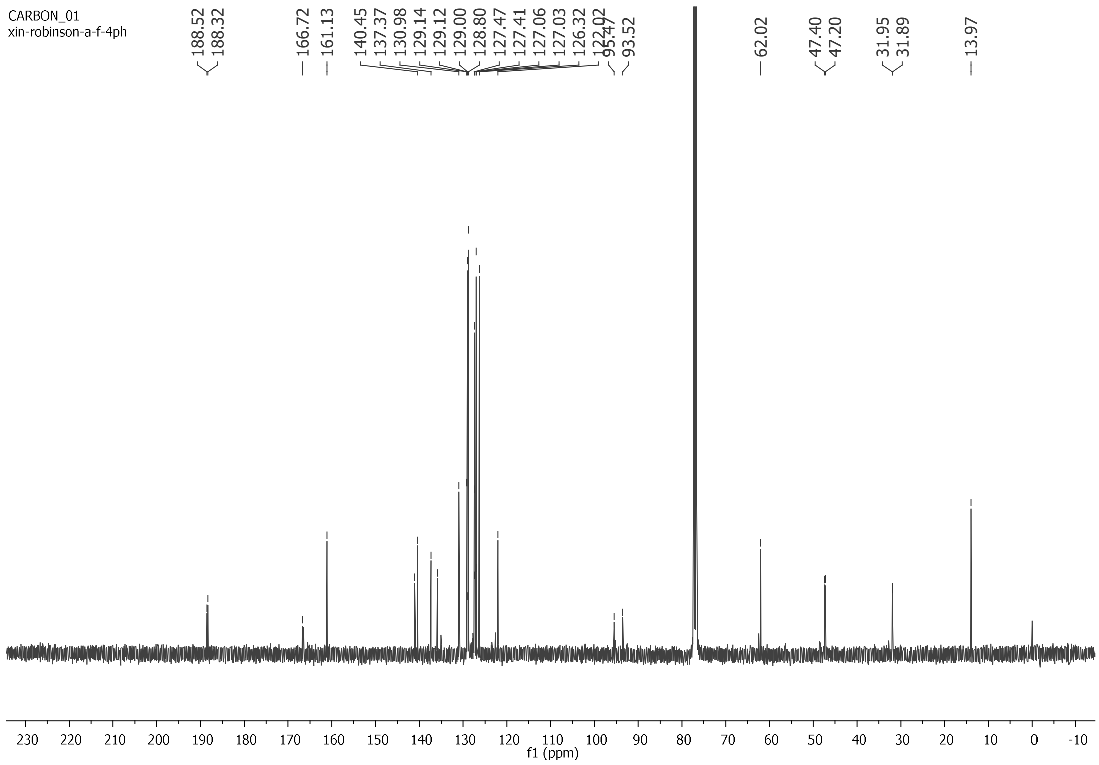
CARBON_01
xin-robinson-a-f-4ph
188.52
188.32
166.72
161.13
140.45
137.37
130.98
129.14
129.12
129.00
128.80
127.47
127.41
127.06
127.03
126.32
93.52
62.02
47.40
47.20
31.95
31.89
13.97
f1 (ppm)

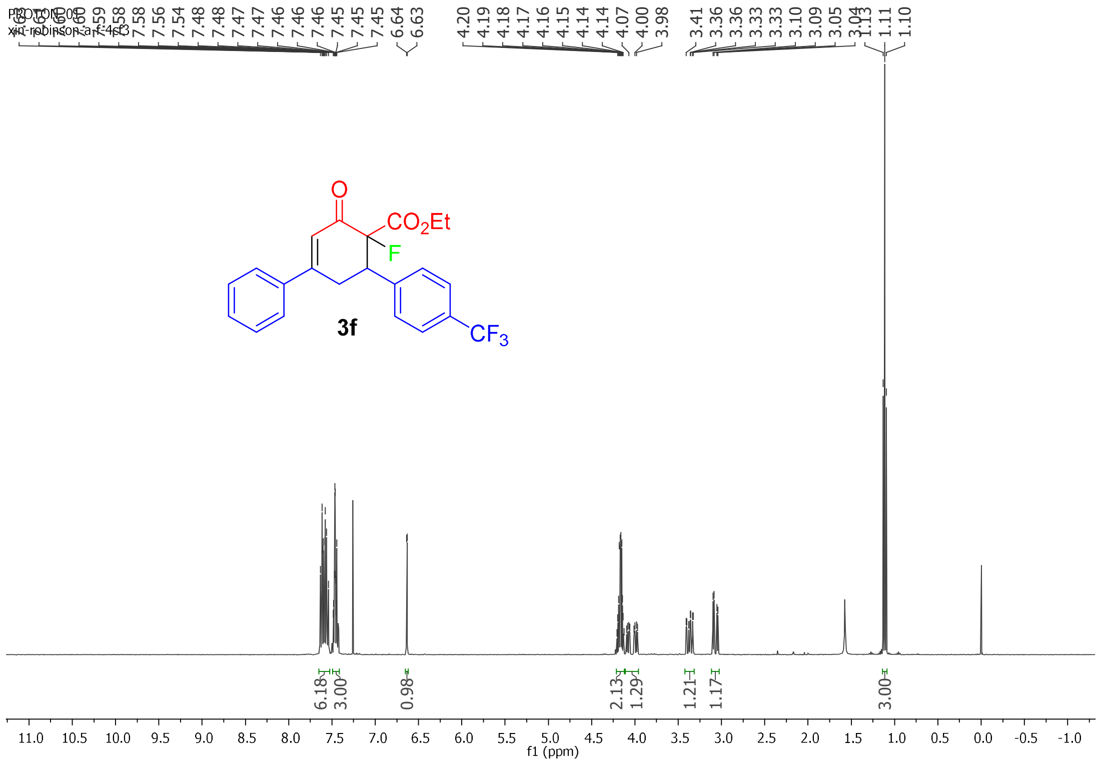
7.58
7.56
7.54
7.48
7.48
7.47
7.47
7.46
7.46
7.45
7.45
7.45
6.64
6.63
4.20
4.19
4.18
4.17
4.16
4.15
4.14
4.14
4.07
4.00
3.98
3.41
3.36
3.36
3.33
3.33
3.10
3.09
3.05
1.13
1.11
1.10
CO2Et
F
CF3
3f
6.18
3.00
0.98
2.13
1.29
1.21
1.17
3.00
f1 (ppm)

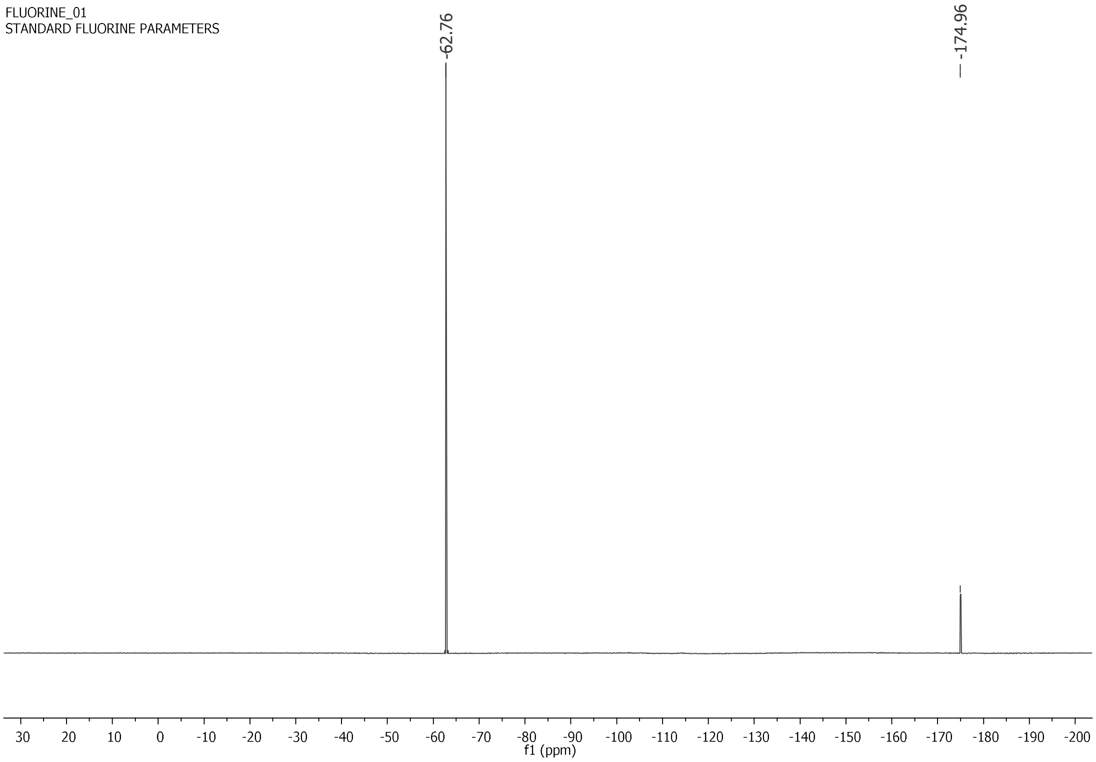
FLUORINE_01
STANDARD FLUORINE PARAMETERS
-62.76
-174.96
30 20 10 0 -10 -20 -30 -40 -50 -60 -70 -80 -90 -100 -110 -120 -130 -140 -150 -160 -170 -180 -190 -200
f1 (ppm)

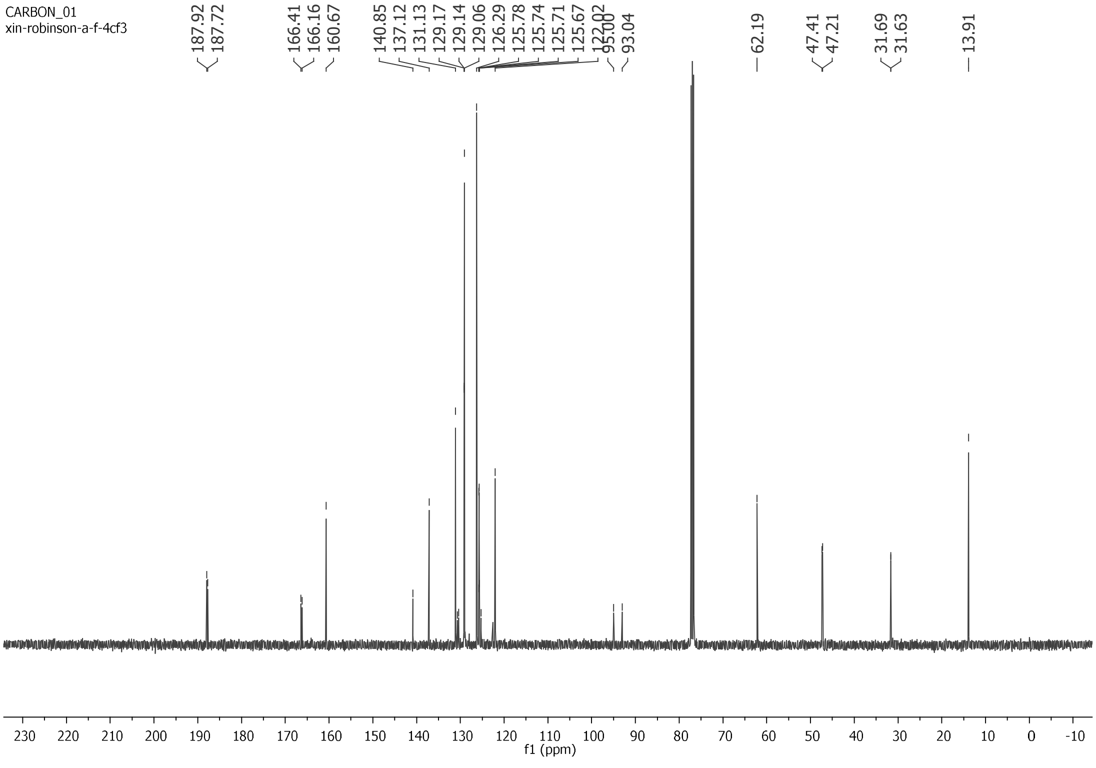
CARBON_01
xin-robinson-a-f-4cf3
187.92
187.72
166.41
166.16
160.67
140.85
137.12
131.13
129.17
129.14
129.06
126.29
125.78
125.74
125.71
125.67
122.02
95.00
93.04
62.19
47.41
47.21
31.69
31.63
13.91
230 220 210 200 190 180 170 160 150 140 130 120 110 100 90 80 70 60 50 40 30 20 10 0 -10
f1 (ppm)

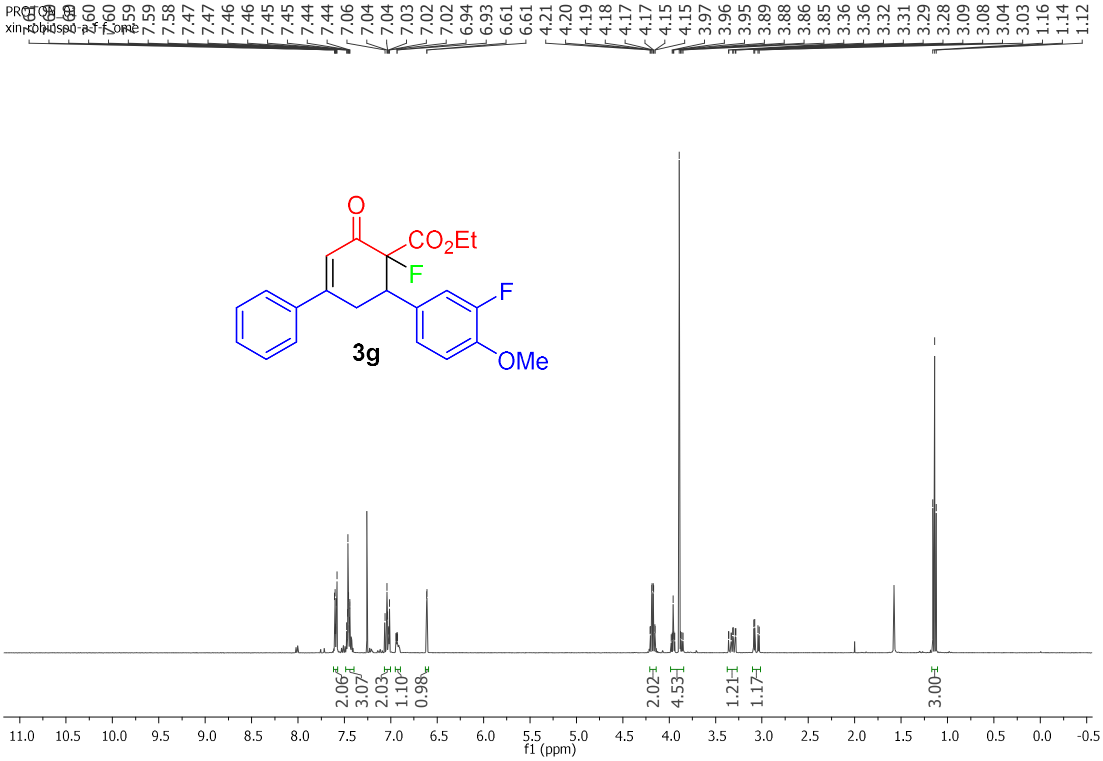
PROTON_01
xin-robinson-a-f-f_ome
7.60
7.59
7.59
7.58
7.47
7.46
7.46
7.45
7.45
7.44
7.44
7.06
7.04
7.04
7.03
7.02
7.02
6.94
6.93
6.61
6.61
4.21
4.20
4.19
4.18
4.17
4.17
4.15
4.15
3.97
3.96
3.95
3.89
3.88
3.86
3.85
3.36
3.36
3.32
3.31
3.29
3.28
3.09
3.08
3.04
3.03
1.16
1.14
1.12
O
CO_2Et
F
F
OMe
3g
2.06
3.07
2.03
1.10
0.98
2.02
4.53
1.21
1.17
3.00
f1 (ppm)

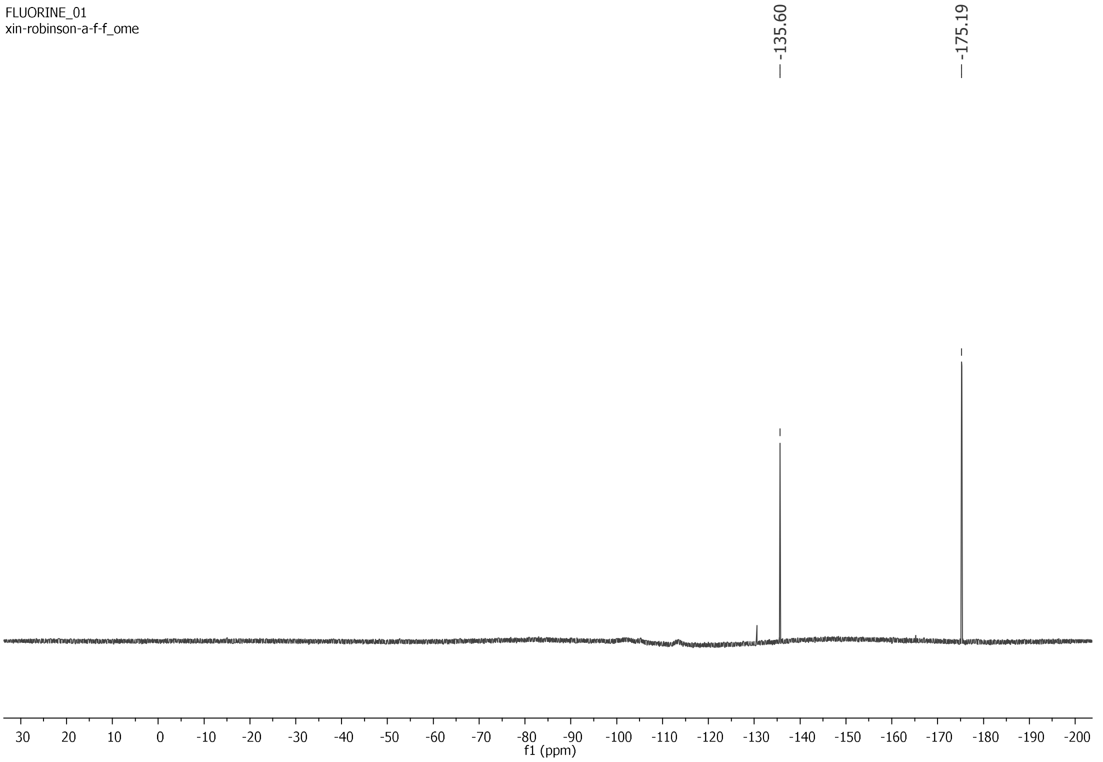
FLUORINE_01
xin-robinson-a-f-f_ome
-135.60
-175.19
f1 (ppm)

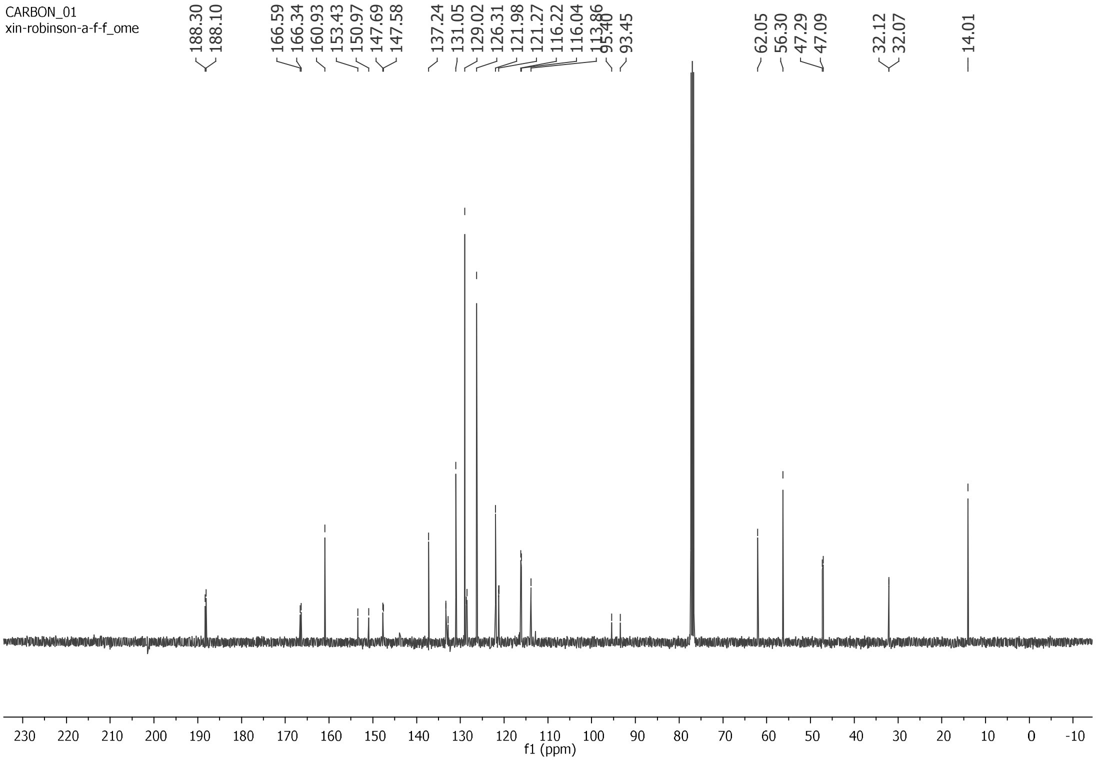
CARBON_01
xin-robinson-a-f-f_ome
188.30
188.10
166.59
166.34
160.93
153.43
150.97
147.69
147.58
137.24
131.05
129.02
126.31
121.98
121.27
116.22
116.04
113.86
95.40
93.45
62.05
56.30
47.29
47.09
32.12
32.07
14.01
230 220 210 200 190 180 170 160 150 140 130 120 110 100 90 80 70 60 50 40 30 20 10 0 -10
f1 (ppm)

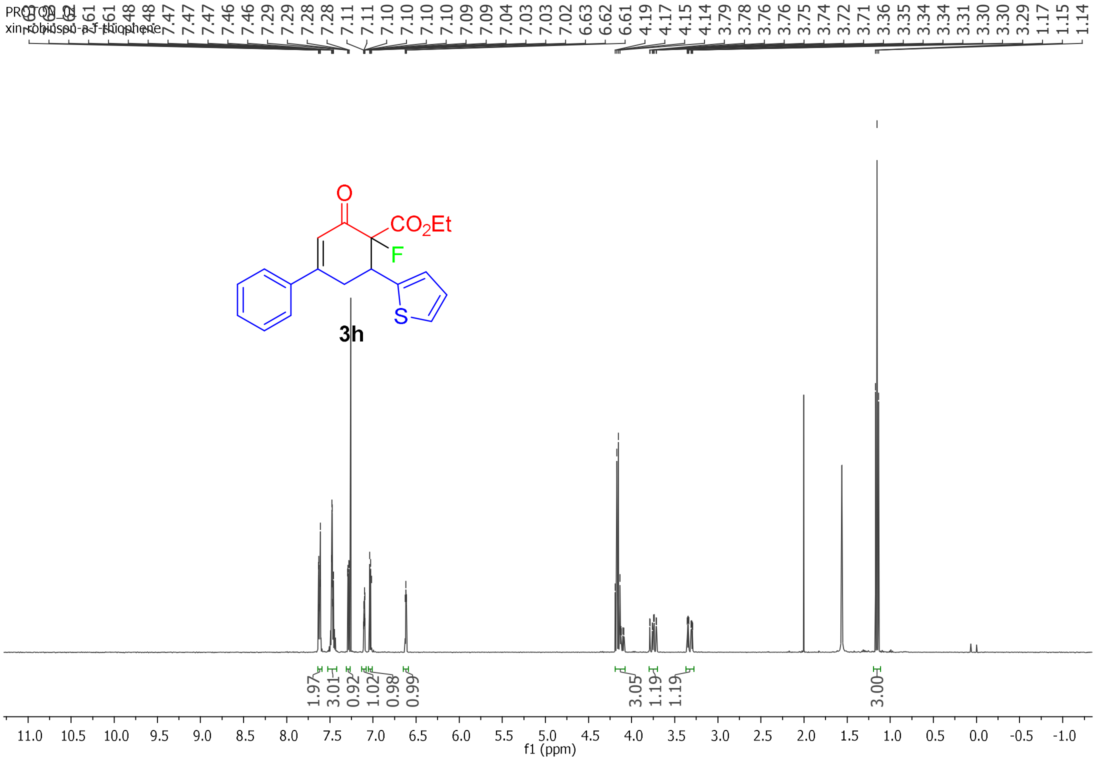
PROTON_01
xin-robinson-a-f-thiophene
7.61
7.48
7.48
7.47
7.47
7.46
7.46
7.29
7.29
7.28
7.28
7.11
7.11
7.10
7.10
7.10
7.10
7.09
7.04
7.03
7.03
7.02
6.63
6.62
6.61
4.19
4.17
4.15
4.14
3.79
3.78
3.76
3.75
3.74
3.72
3.71
3.36
3.35
3.34
3.34
3.31
3.30
3.30
3.29
1.17
1.15
1.14
O
CO2Et
F
S
3h
1.97
3.01
0.92
1.02
0.98
0.99
3.05
1.19
1.19
3.00
11.0 10.5 10.0 9.5 9.0 8.5 8.0 7.5 7.0 6.5 6.0 5.5 5.0 4.5 4.0 3.5 3.0 2.5 2.0 1.5 1.0 0.5 0.0 -0.5 -1.0
f1 (ppm)

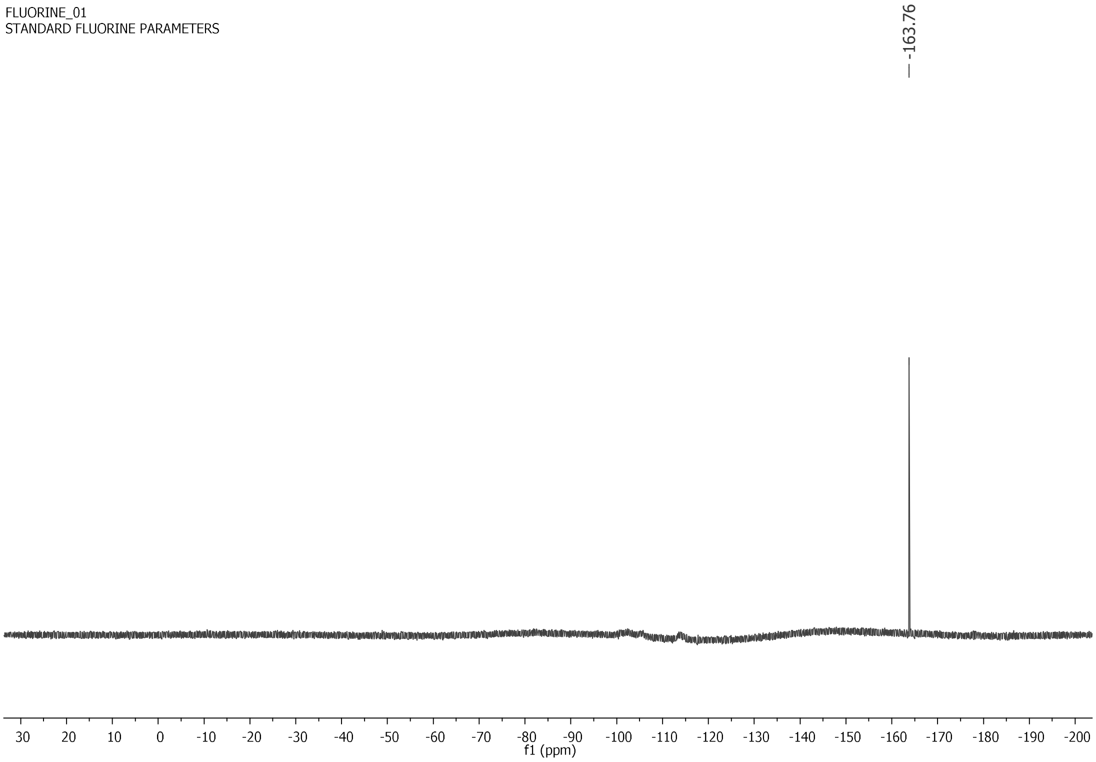

FLUORINE_01
STANDARD FLUORINE PARAMETERS
-163.76
30 20 10 0 -10 -20 -30 -40 -50 -60 -70 -80 -90 -100 -110 -120 -130 -140 -150 -160 -170 -180 -190 -200
f1 (ppm)

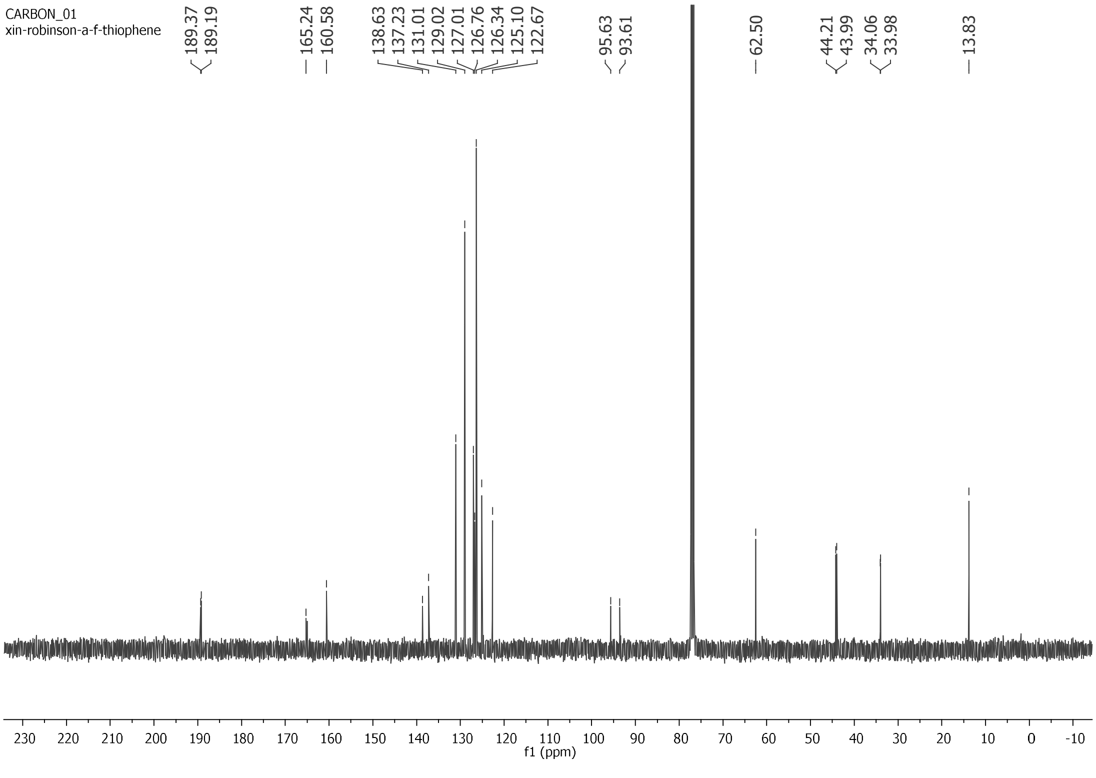

CARBON_01
xin-robinson-a-f-thiophene
189.37
189.19
165.24
160.58
138.63
137.23
131.01
129.02
127.01
126.76
126.34
125.10
122.67
95.63
93.61
62.50
44.21
43.99
34.06
33.98
13.83
230 220 210 200 190 180 170 160 150 140 130 120 110 100 90 80 70 60 50 40 30 20 10 0 -10
f1 (ppm)

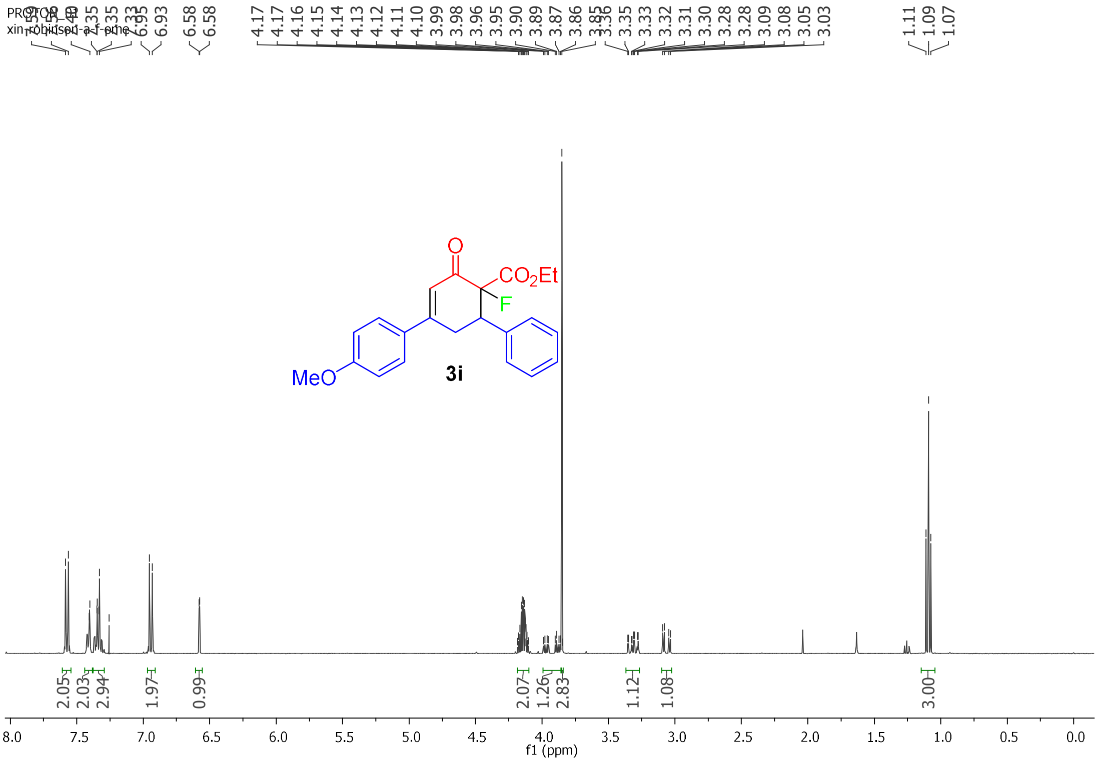
CO2Et
F
MeO
3i
f1 (ppm)

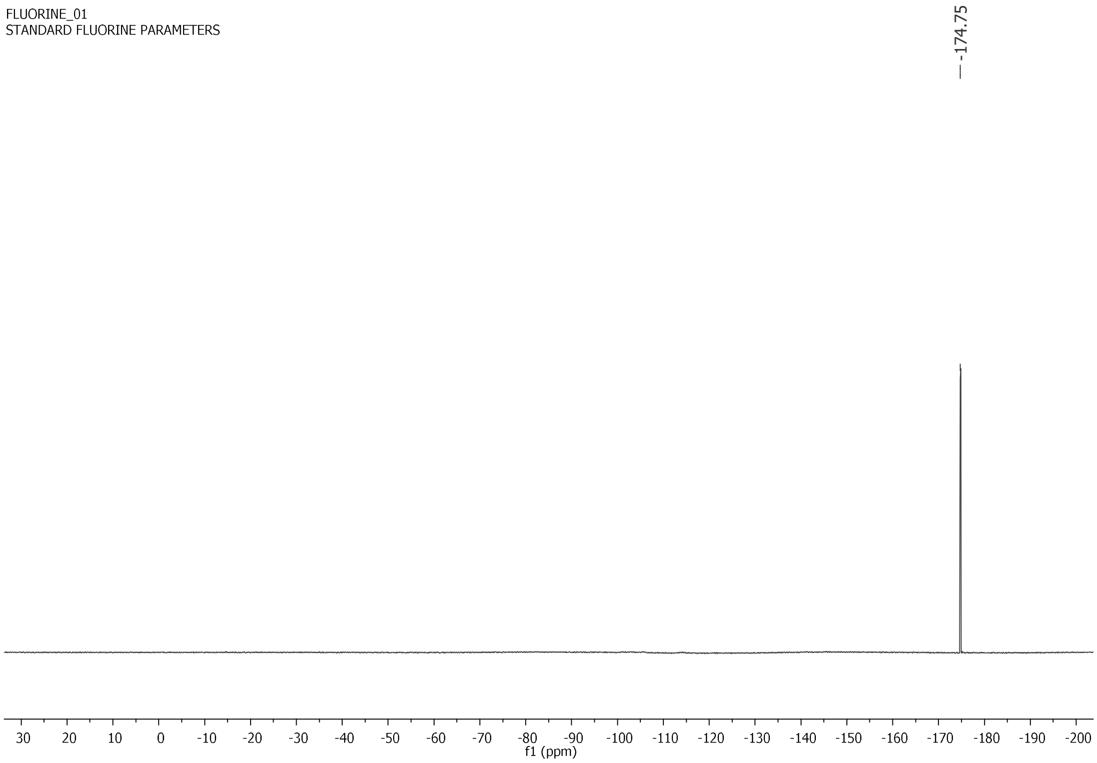
FLUORINE_01
STANDARD FLUORINE PARAMETERS
-174.75
f1 (ppm)

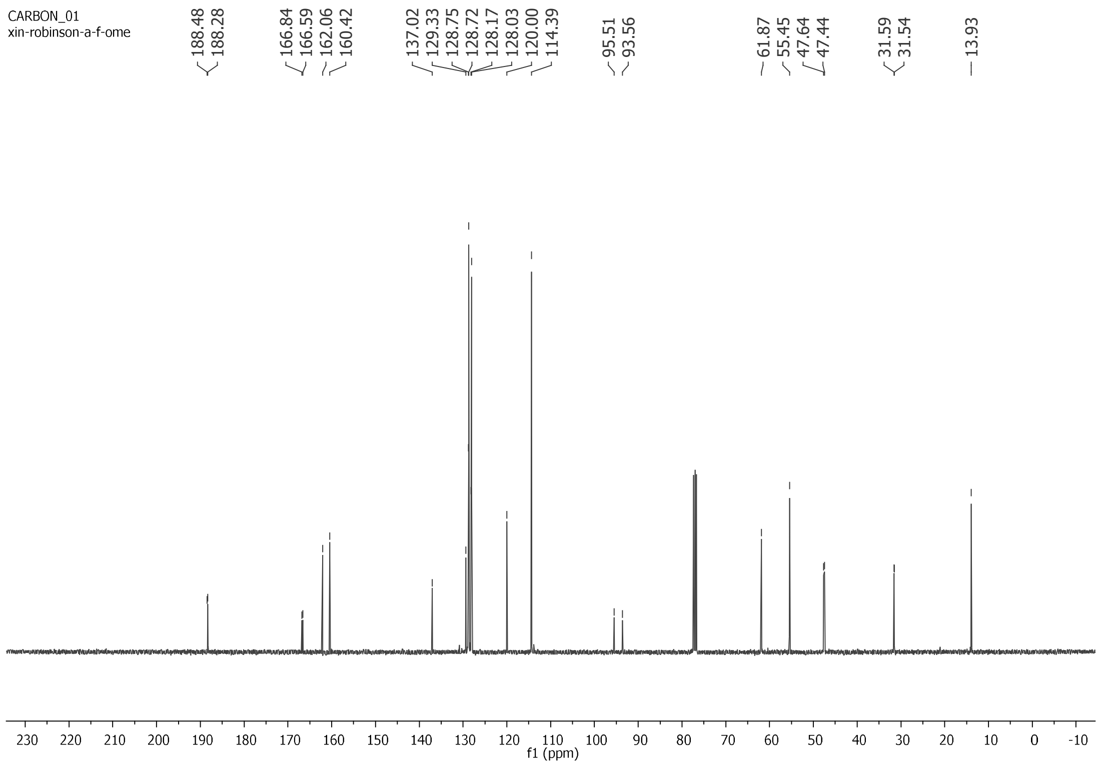
CARBON_01
xin-robinson-a-f-ome
188.48
188.28
166.84
166.59
162.06
160.42
137.02
129.33
128.75
128.72
128.17
128.03
120.00
114.39
95.51
93.56
61.87
55.45
47.64
47.44
31.59
31.54
13.93
230 220 210 200 190 180 170 160 150 140 130 120 110 100 90 80 70 60 50 40 30 20 10 0 -10
f1 (ppm)

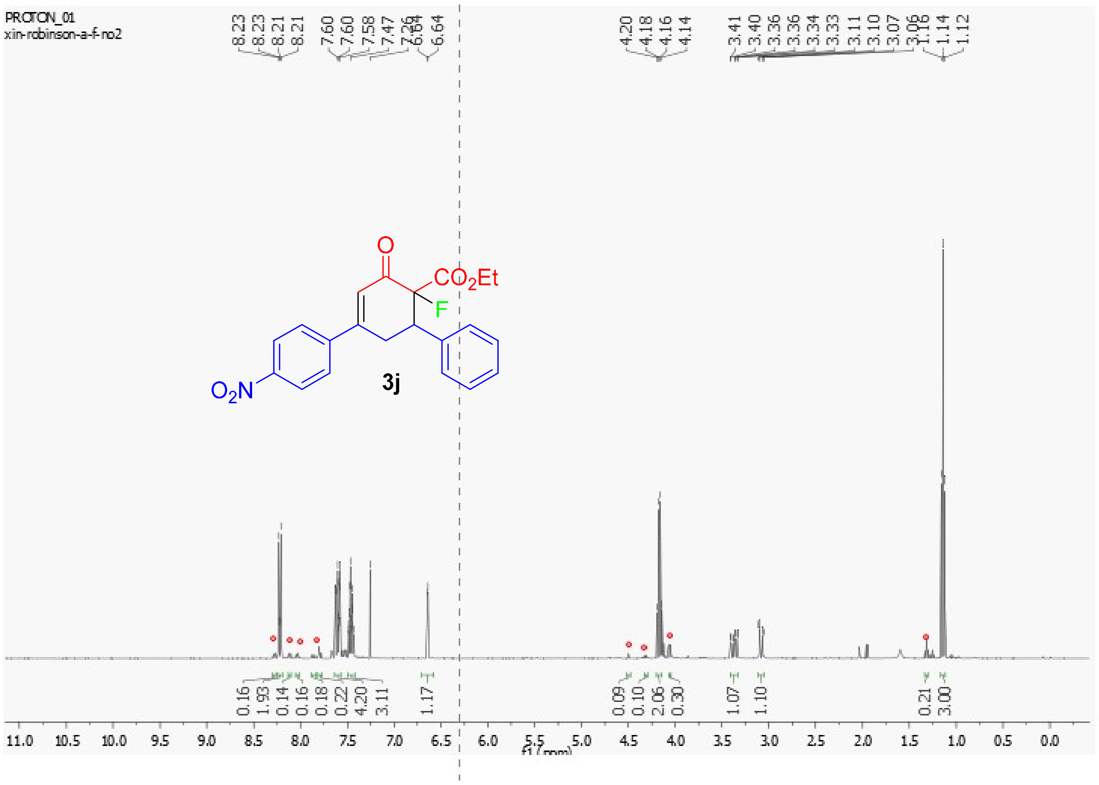
PROTON_01
xin-robinson-a-f-no2
8.23
8.23
8.21
8.21
7.60
7.60
7.58
7.47
7.26
6.64
6.64
4.20
4.18
4.16
4.14
3.41
3.40
3.36
3.36
3.34
3.33
3.11
3.10
3.07
3.06
1.16
1.14
1.12
O
CO2Et
F
O2N
3j
0.16
1.93
0.14
0.16
0.18
0.22
4.20
3.11
1.17
0.09
0.10
2.06
0.30
1.07
1.10
0.21
3.00
11.0 10.5 10.0 9.5 9.0 8.5 8.0 7.5 7.0 6.5 6.0 5.5 5.0 4.5 4.0 3.5 3.0 2.5 2.0 1.5 1.0 0.5 0.0
f1 (ppm)

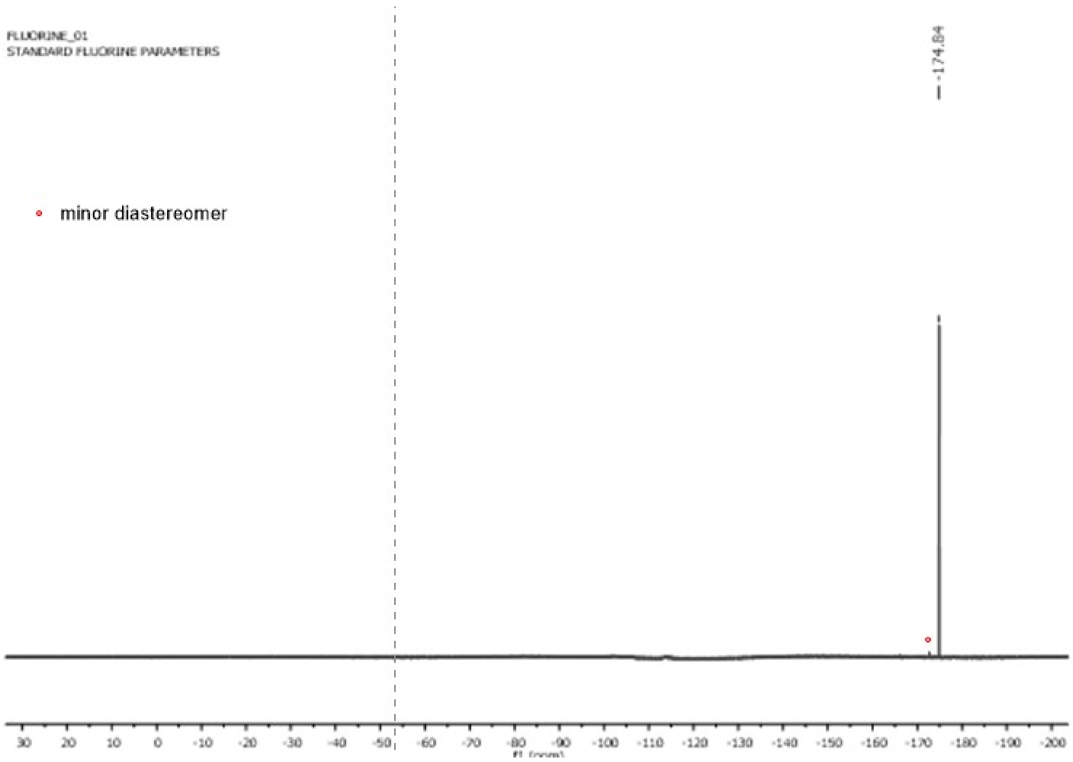
FLUORINE_01
STANDARD FLUORINE PARAMETERS
-174.84
minor diastereomer
f1 (ppm)

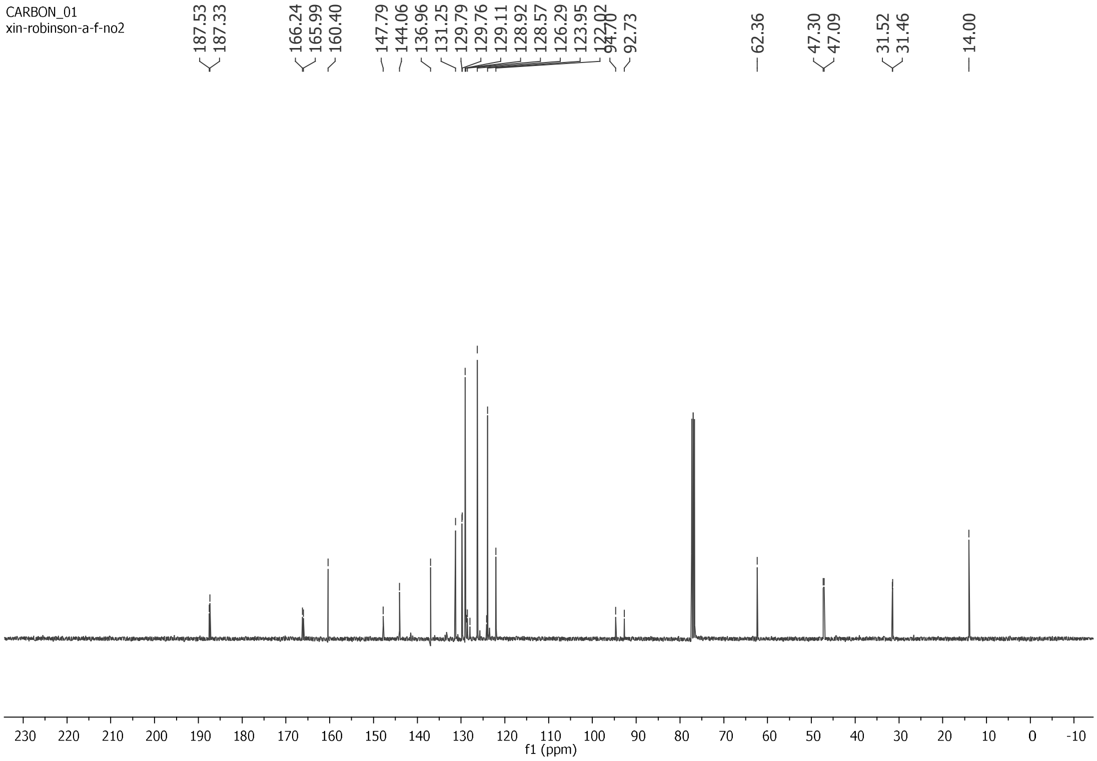
CARBON_01
xin-robinson-a-f-no2
187.53
187.33
166.24
165.99
160.40
147.79
144.06
136.96
131.25
129.79
129.76
129.11
128.92
128.57
126.29
123.95
122.02
94.70
92.73
62.36
47.30
47.09
31.52
31.46
14.00
f1 (ppm)

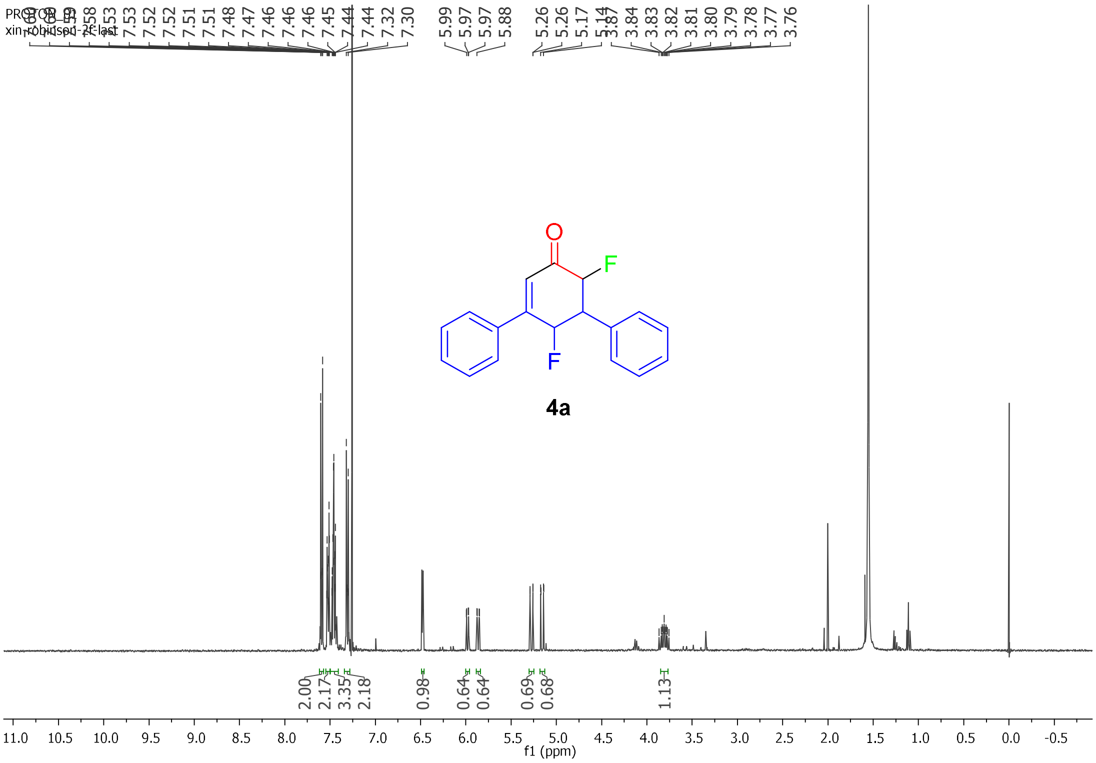

7.53
7.53
7.52
7.52
7.51
7.51
7.48
7.47
7.46
7.46
7.46
7.45
7.44
7.44
7.32
7.30
5.99
5.97
5.97
5.88
5.26
5.26
5.17
5.14
3.84
3.83
3.82
3.81
3.80
3.79
3.78
3.77
3.76
O
F
F
4a
2.00
2.17
3.35
2.18
0.98
0.64
0.64
0.69
0.68
1.13
f1 (ppm)

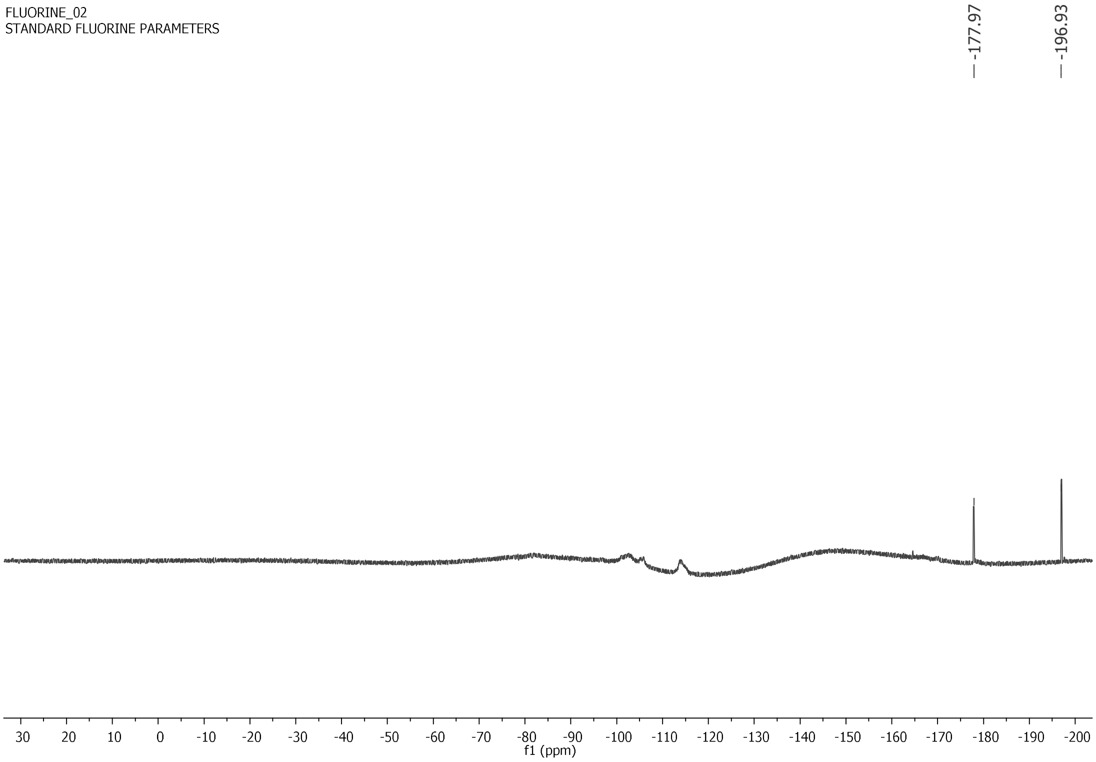

FLUORINE_02
STANDARD FLUORINE PARAMETERS
-177.97
-196.93
f1 (ppm)

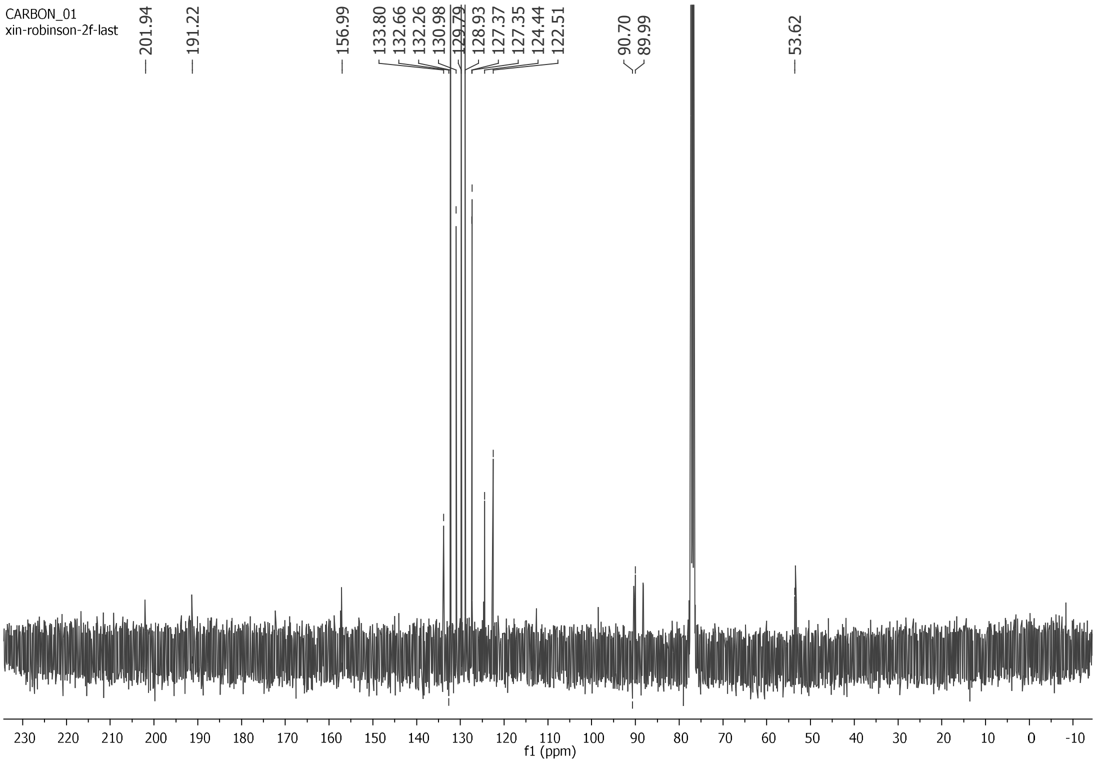
CARBON_01
xin-robinson-2f-last
201.94
191.22
156.99
133.80
132.66
132.26
130.98
129.79
128.93
127.37
127.35
124.44
122.51
90.70
89.99
53.62
f1 (ppm)

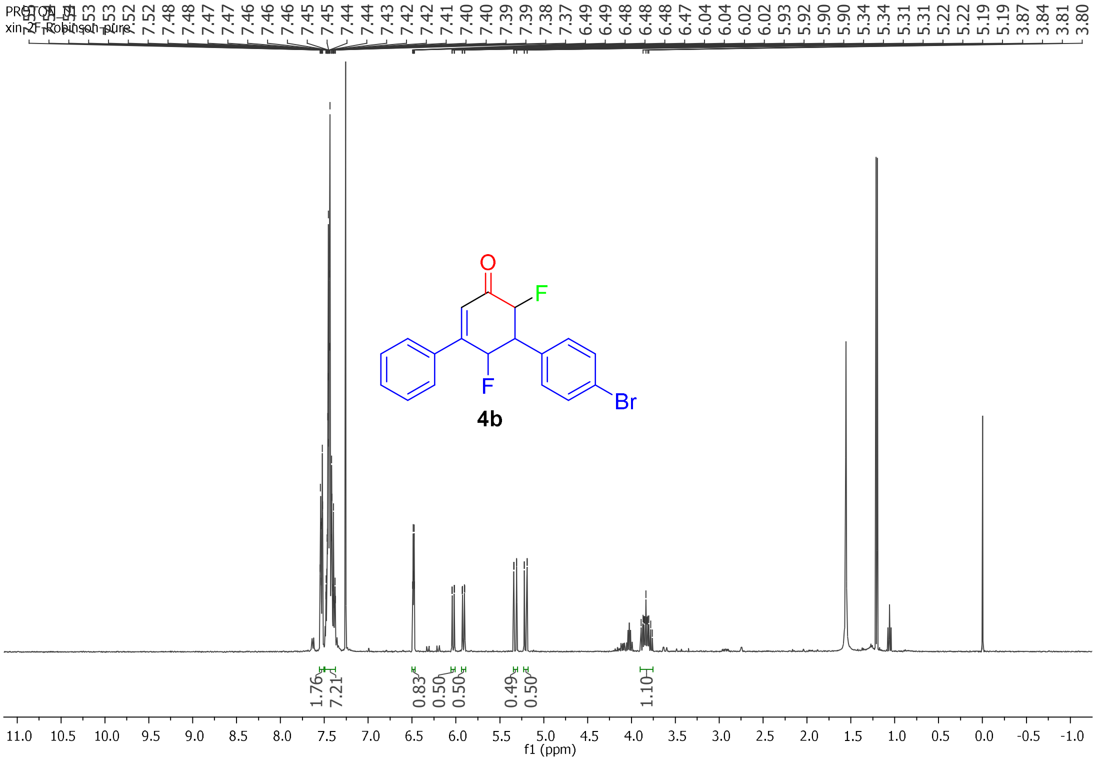
7.53
7.53
7.52
7.48
7.48
7.47
7.47
7.46
7.46
7.46
7.45
7.45
7.44
7.44
7.43
7.42
7.42
7.41
7.40
7.40
7.39
7.39
7.38
7.37
6.49
6.49
6.48
6.48
6.48
6.47
6.04
6.04
6.02
6.02
5.93
5.92
5.90
5.90
5.34
5.34
5.31
5.31
5.22
5.22
5.19
5.19
3.87
3.84
3.81
3.80
O
F
F
Br
4b
1.76
7.21
0.83
0.50
0.50
0.49
0.50
1.10
f1 (ppm)

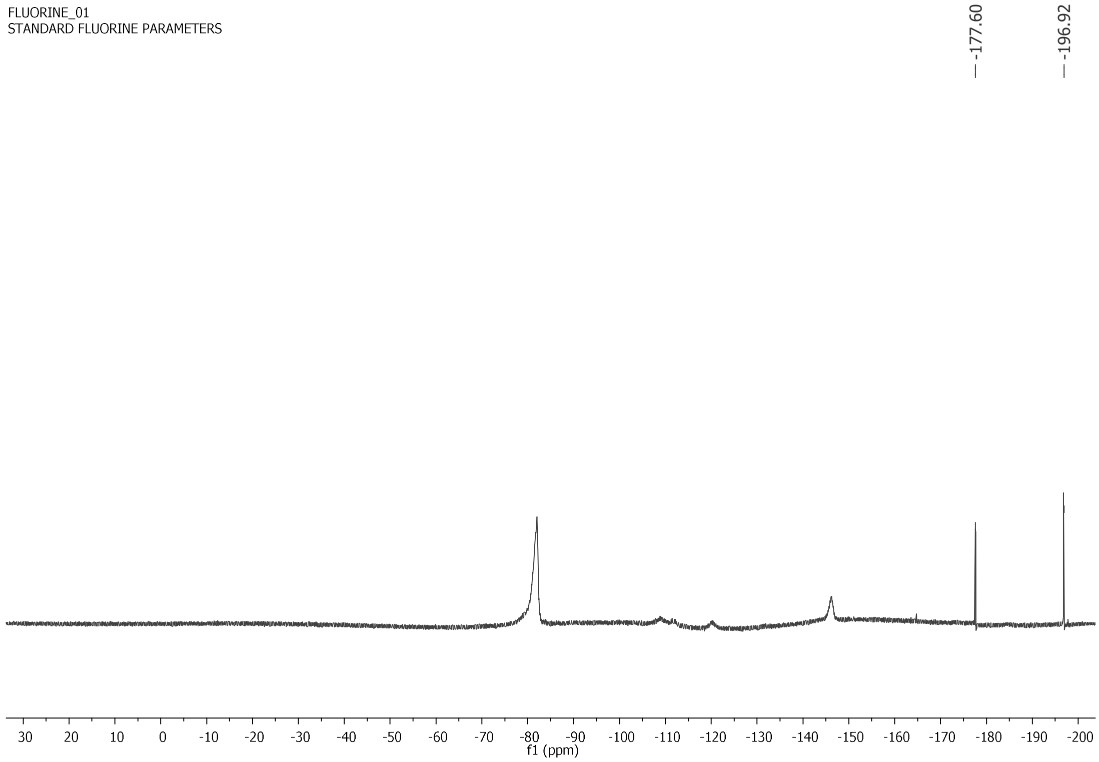
FLUORINE_01
STANDARD FLUORINE PARAMETERS
-177.60
-196.92
f1 (ppm)

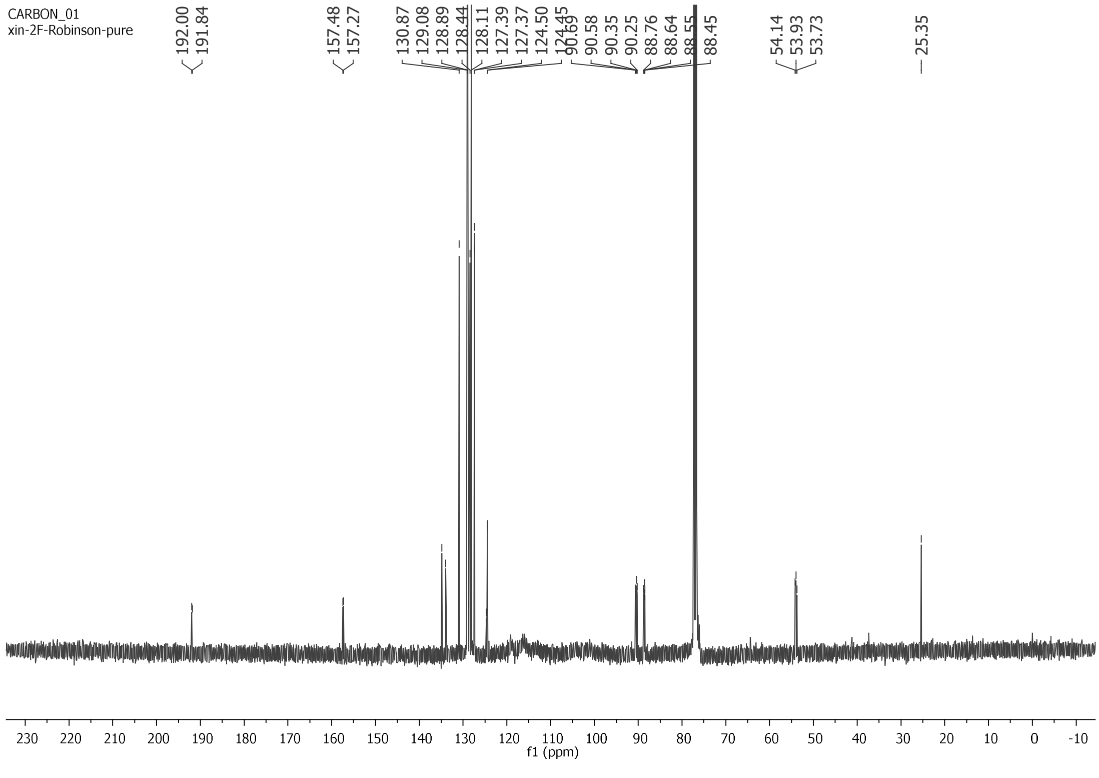
CARBON_01
xin-2F-Robinson-pure
192.00
191.84
157.48
157.27
130.87
129.08
128.89
128.44
128.11
127.39
127.37
124.50
124.45
90.69
90.58
90.35
90.25
88.76
88.64
88.55
88.45
54.14
53.93
53.73
25.35
f1 (ppm)

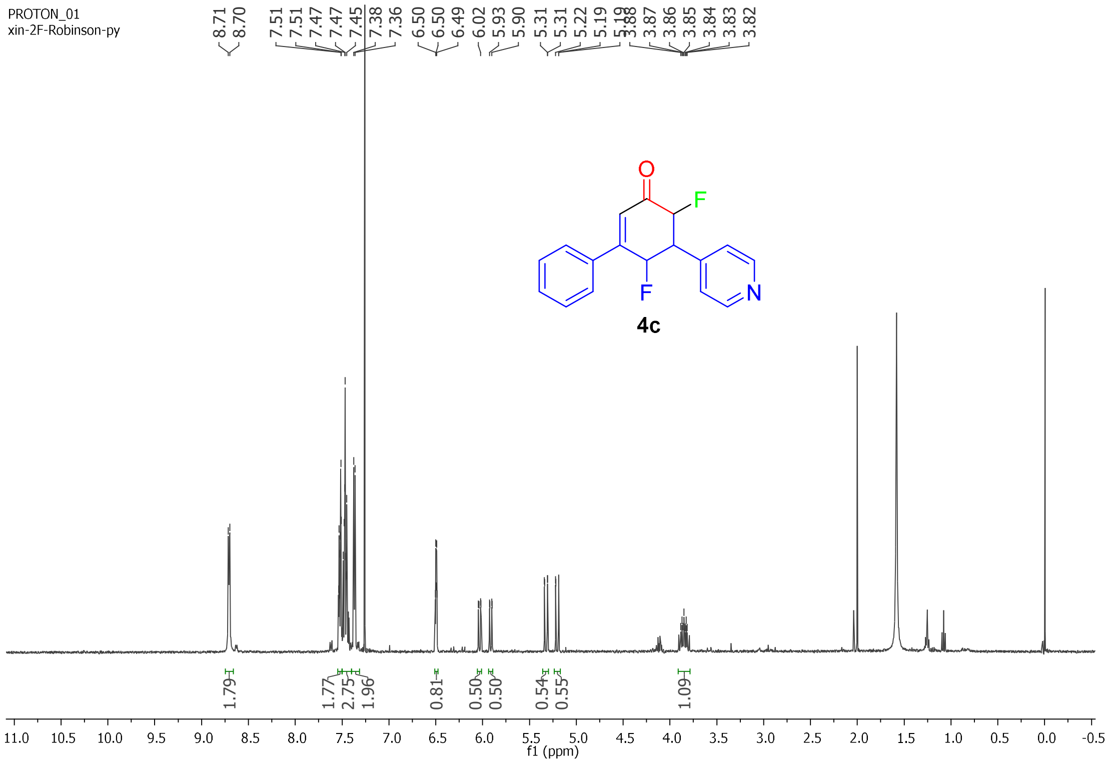
PROTON_01
xin-2F-Robinson-py
8.71
8.70
7.51
7.51
7.47
7.47
7.45
7.38
7.36
6.50
6.50
6.49
6.02
5.93
5.90
5.31
5.31
5.22
5.19
5.19
3.88
3.87
3.86
3.85
3.84
3.83
3.82
O
F
F
N
4c
1.79
1.77
2.75
1.96
0.81
0.50
0.50
0.54
0.55
1.09
11.0 10.5 10.0 9.5 9.0 8.5 8.0 7.5 7.0 6.5 6.0 5.5 5.0 4.5 4.0 3.5 3.0 2.5 2.0 1.5 1.0 0.5 0.0 -0.5
f1 (ppm)

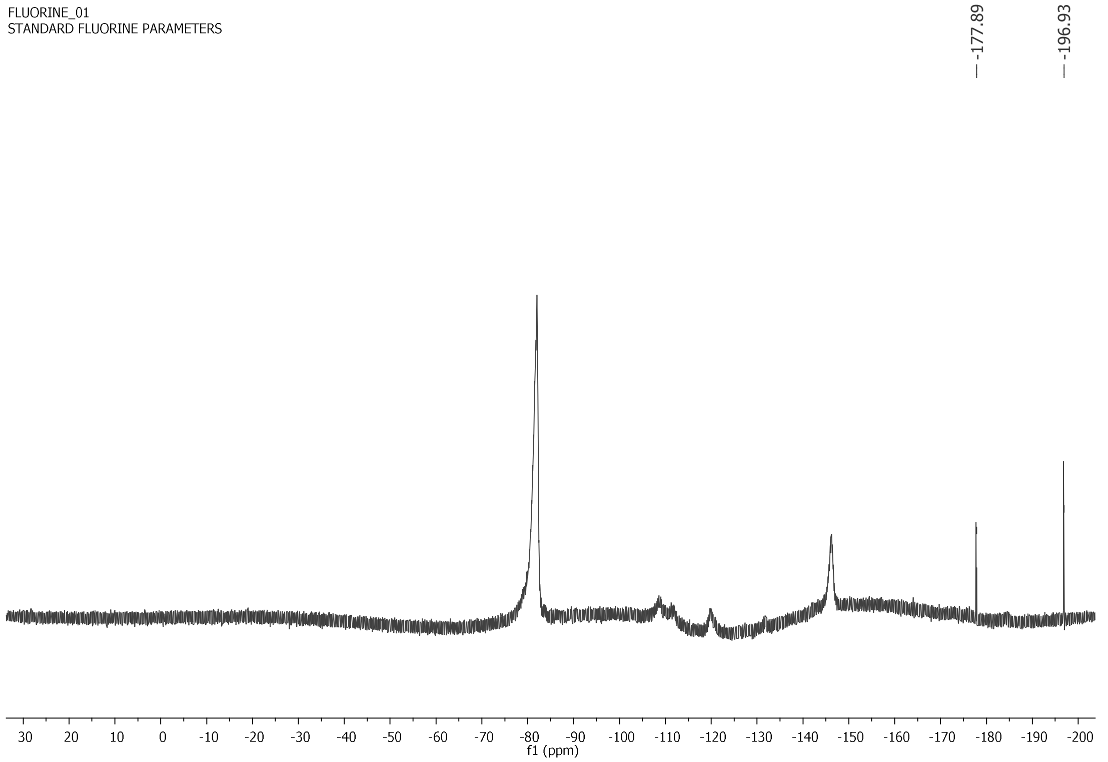
FLUORINE_01
STANDARD FLUORINE PARAMETERS
-177.89
-196.93
30 20 10 0 -10 -20 -30 -40 -50 -60 -70 -80 -90 -100 -110 -120 -130 -140 -150 -160 -170 -180 -190 -200
f1 (ppm)

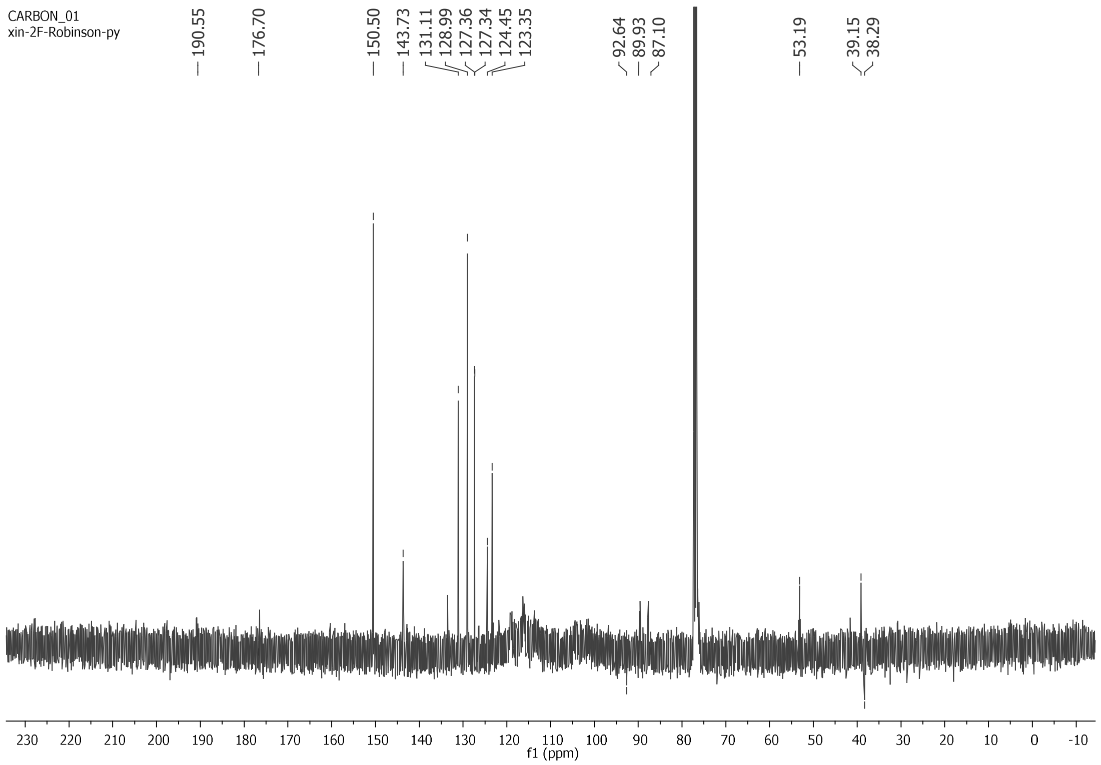
CARBON_01
xin-2F-Robinson-py
190.55
176.70
150.50
143.73
131.11
128.99
127.36
127.34
124.45
123.35
92.64
89.93
87.10
53.19
39.15
38.29
230 220 210 200 190 180 170 160 150 140 130 120 110 100 90 80 70 60 50 40 30 20 10 0 -10
f1 (ppm)

4. Chiral LC of Products

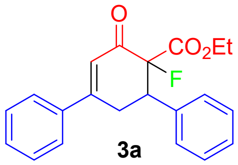

3a

Racemic sample

Retention	Area %	Height %
6.312	11.35	17.12
7.484	11.37	14.00
9.492	38.68	37.50
12.084	38.61	31.38
Totals	100.00	100.00

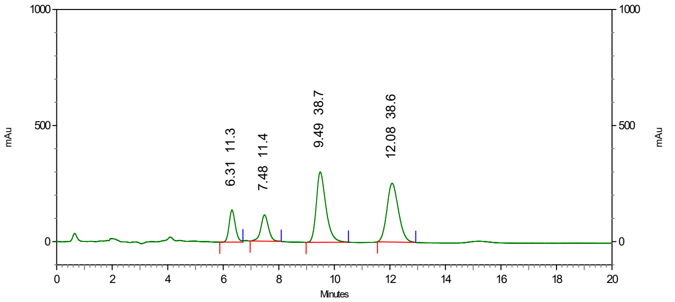

Enantioenriched sample

Retention	Area %	Height %
6.268	5.80	9.54
7.136	0.38	0.57
9.376	93.28	89.35
12.152	0.55	0.54
Totals	100.00	100.00

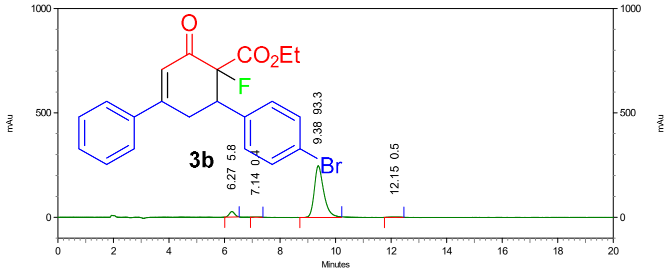

Racemic sample

Retention	Area %	Height %
11.552	33.19	45.82
14.588	33.00	26.23
17.700	16.99	17.34

26.384	16.81	10.61
Totals	100.00	100.00

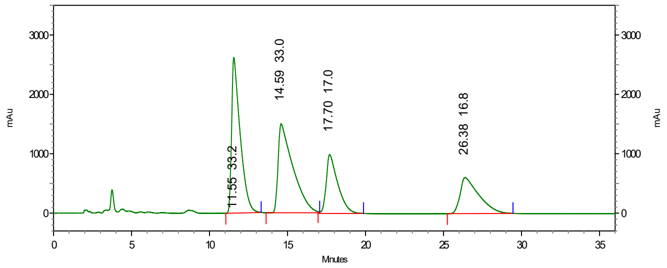

Enantioenriched sample

Retention	Area %	Height %
11.743	100.00	10.00
Totals	100.00	100.00

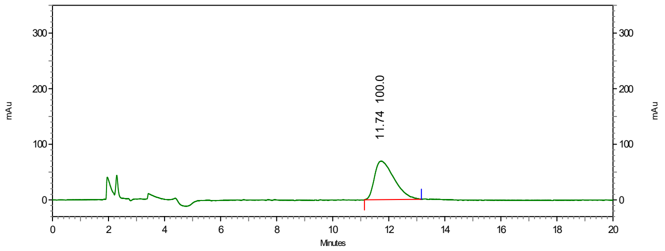

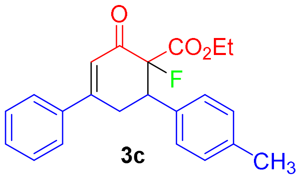

3c

Racemic sample

Retention	Area %	Height %
15.096	39.39	45.80
16.228	41.84	38.98
20.436	9.48	7.86
22.988	9.29	7.36
Totals	100.00	100.00

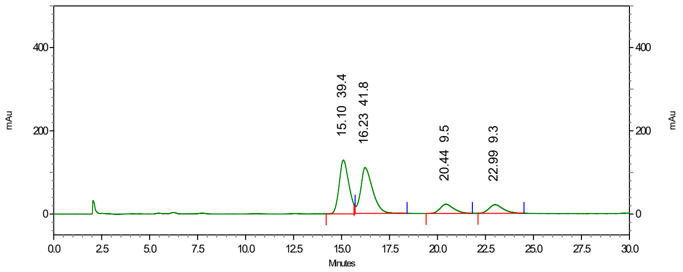

Enantioenriched sample

Retention	Area %	Height %
15.340	1.92	2.96

16.240	98.08	97.04
Totals	100.00	100.00

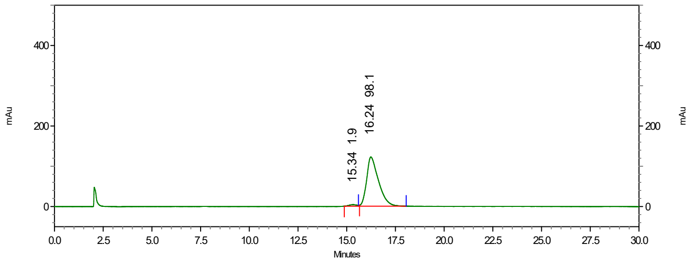

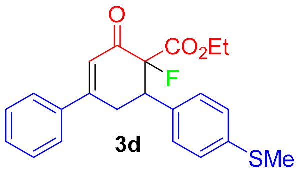

3d

Racemic sample

Retention	Area %	Height %
5.408	11.61	16.22
6.388	11.27	12.83
7.144	38.65	39.87
9.024	38.48	31.07
Totals	100.00	100.00

Retention	Area %	Height %
5.396	9.60	12.41
6.120	0.97	1.12
7.112	87.91	85.04
9.052	1.51	1.43
Totals	100.00	100.00

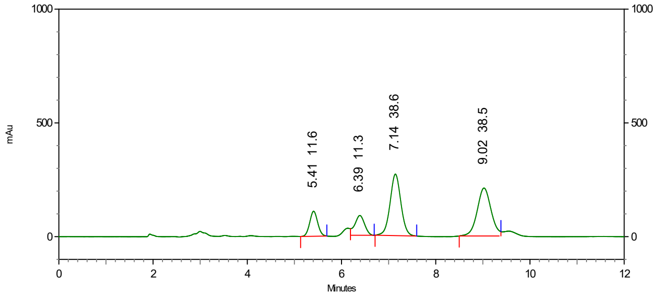

Enantioenriched sample

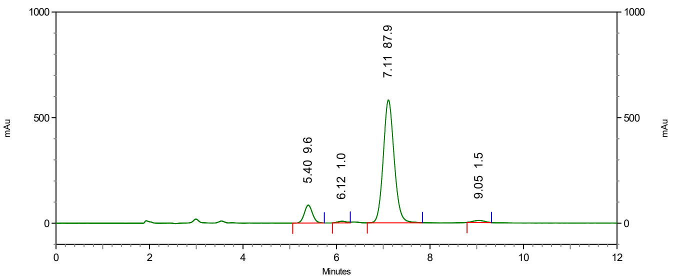

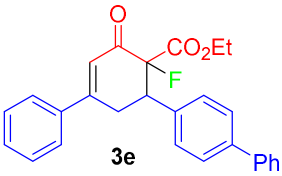

3e

Racemic sample

Retention	Area %	Height %
10.568	49.84	58.43
14.248	50.16	41.57
Totals	100.00	100.00

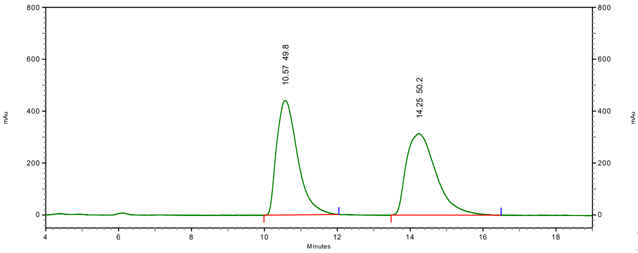

Enantioenriched sample

Retention	Area %	Height %
10.604	96.52	97.31
15.576	3.48	2.69
Totals	100.00	100.00

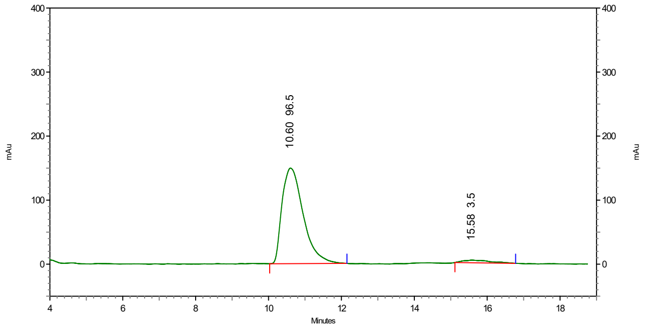

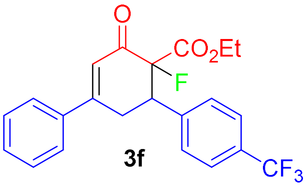

3f

Racemic sample

Retention	Area %	Height %
13.772	50.91	55.65
19.428	49.09	44.35
Totals	100.00	100.00

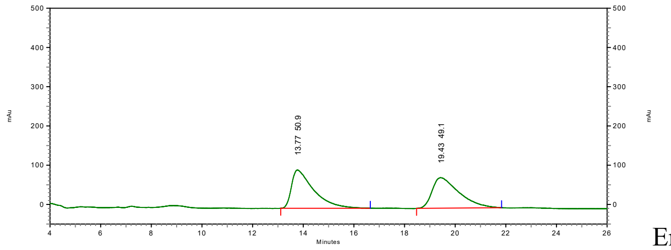

Enantioenriched sample

Retention	Area %	Height %
13.968	100.00	100.00
Totals	100.00	100.00

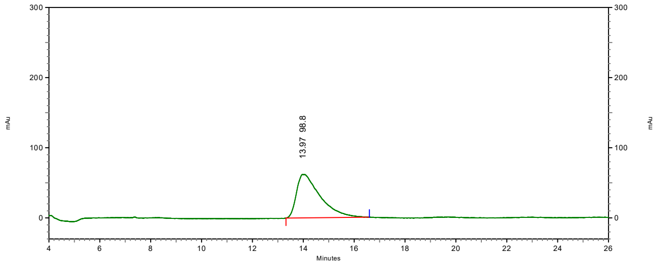

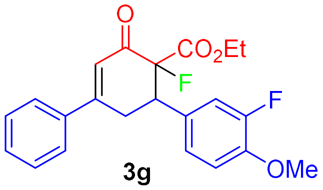

3g

Racemic sample

Retention	Area %	Height %
3.212	33.35	39.53
3.504	33.89	34.83
4.840	16.40	13.45
5.696	16.35	12.19
Totals	100.00	100.00

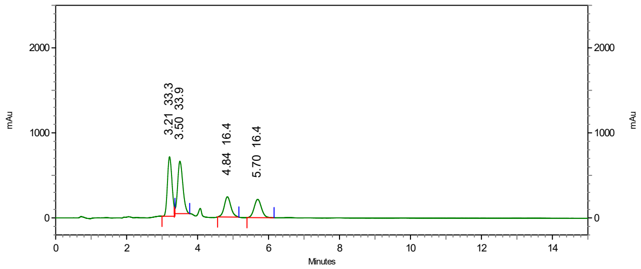

Enantioenriched sample

Retention	Area %	Height %
3.252	3.46	4.30
3.732	96.54	95.70
Totals	100.00	100.00

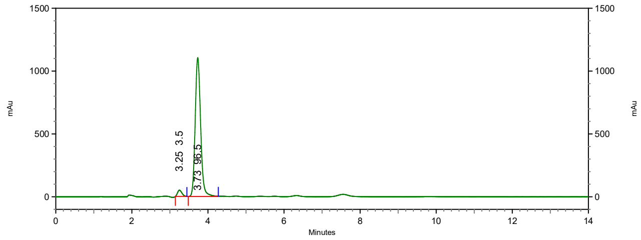

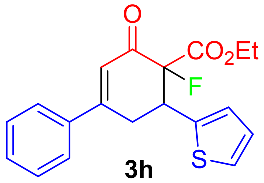

3h

Racemic sample

Retention	Area %	Height %
14.764	7.48	12.71
16.696	42.26	55.03
18.480	7.77	9.85
32.668	42.48	22.41
Totals	100.00	100.00

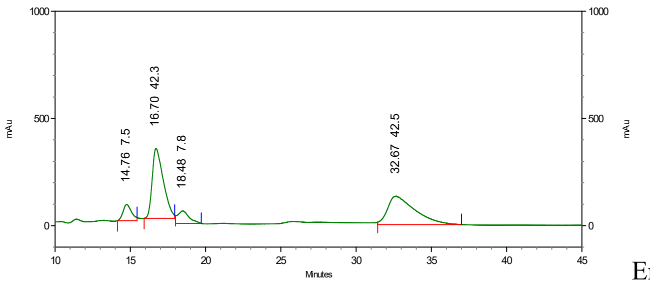

Enantioenriched sample

Retention	Area %	Height %
14.402 (major)	90.62	93.22
17.293 (minor)	3.88	3.83
18.649 (major)	1.21	1.12
34.490 (minor)	4.19	1.83
Totals	100.00	100.00

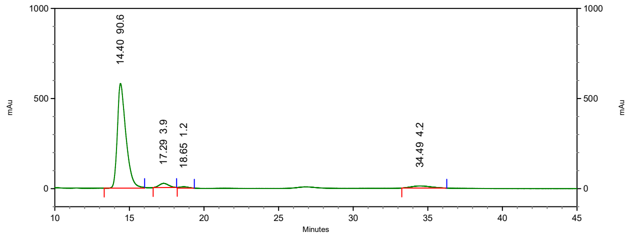

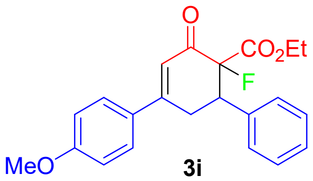

3i

Racemic sample

Retention	Area %	Height %
12.704	49.88	67.54
25.808	50.12	32.46
Totals	100.00	100.00

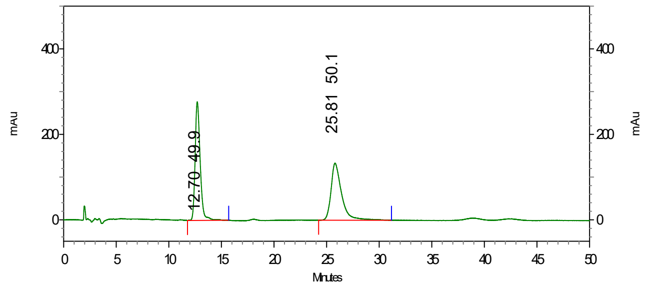

Enantioenriched sample

Retention	Area %	Height %
12.144	94.62	96.17
24.756	5.38	3.83
Totals	100.00	100.00

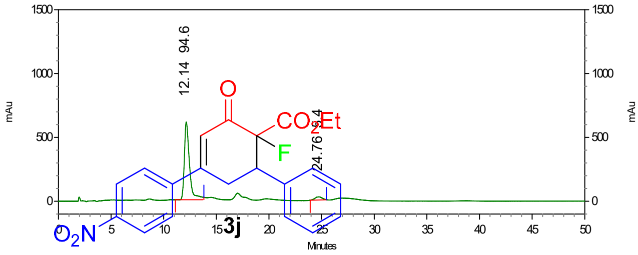

3j

Racemic sample

Retention	Area %	Height %
14.196	49.56	49.90
15.392	50.44	50.10
Totals	100.00	100.00

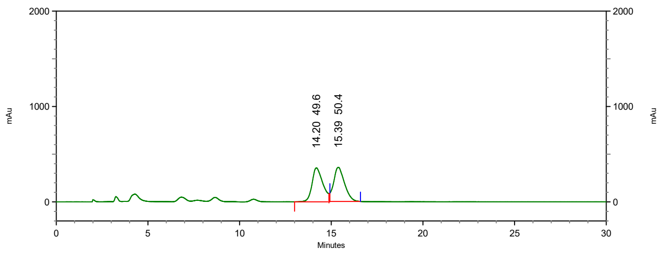

Enantioenriched sample

Retention	Area %	Height %
14.368	92.42	91.57
15.740	7.58	8.43
Totals	100.00	100.00

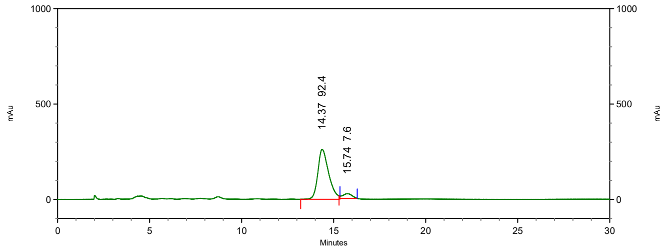

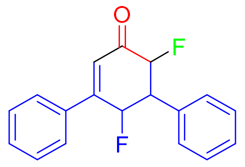

4a

Racemic sample

Retention	Area %	Height %
14.780	50.16	53.33
16.220	49.84	46.67
Totals	100.00	100.00

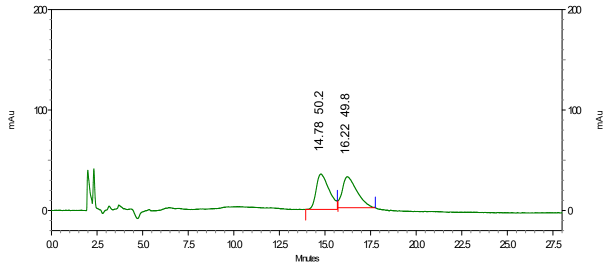

Enantioenriched sample

Retention	Area %	Height %
14.484	96.69	95.59
16.540	3.31	4.41
Totals	100.00	100.00

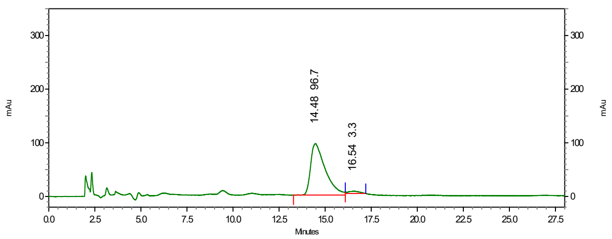

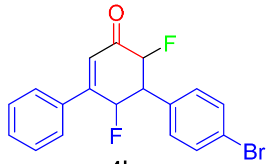

4b

Racemic sample

Retention	Area %	Height %
9.520	50.87	55.40
12.340	49.13	44.60
Totals	100.00	100.00

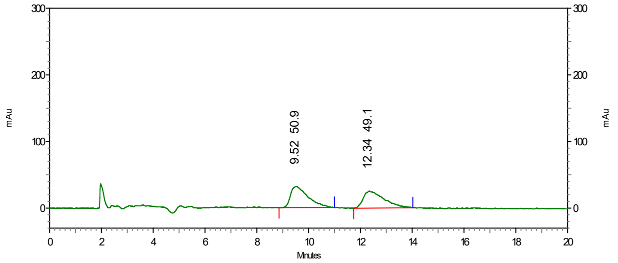

Enantioenriched sample

Retention	Area %	Height %
9.392	94.28	93.10
12.536	5.72	6.90
Totals	100.00	100.00

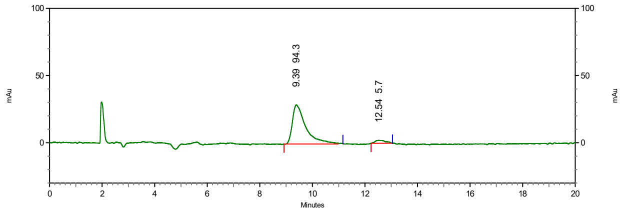

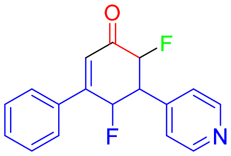

4c

Racemic sample

Retention	Area %	Height %
20.776	51.06	58.25
27.052	48.94	41.75
Totals	100.00	100.00

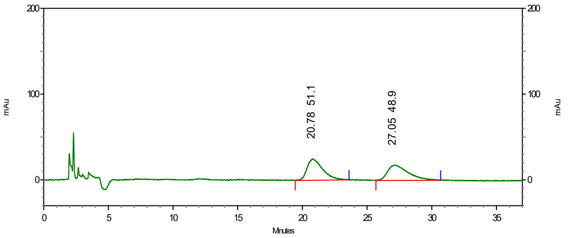

Enantioenriched sample

Retention	Area %	Height %
20.488	96.57	96.19
28.120	3.43	3.81
Totals	100.00	100.00

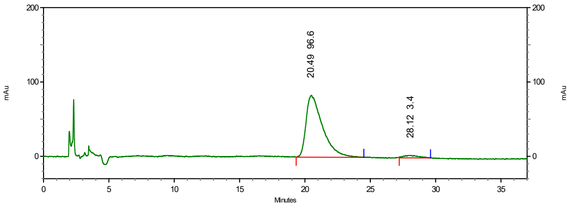

Supplement: Supplementary file 1 [file molecules-23-02251-s001.pdf]
